# Set7 Methyltransferase and Phenotypic Switch in Diabetic Glomerular Endothelial Cells

**DOI:** 10.1681/ASN.0000000000000345

**Published:** 2024-04-17

**Authors:** Scott Maxwell, Jun Okabe, Harikrishnan Kaipananickal, Hanah Rodriguez, Ishant Khurana, Keith Al-Hasani, Bryna S.M. Chow, Eleni Pitsillou, Tom C. Karagiannis, Karin Jandeleit-Dahm, Ronald C.W. Ma, Yu Huang, Juliana C.N. Chan, Mark E. Cooper, Assam El-Osta

**Affiliations:** 1 Epigenetics in Human Health and Disease Laboratory, Baker Heart and Diabetes Institute, Melbourne, Victoria, Australia; 2 Epigenetics in Human Health and Disease Laboratory, Department of Diabetes, Central Clinical School, Monash University, Melbourne, Victoria, Australia; 3 Department of Clinical Pathology, The University of Melbourne, Parkville, Victoria, Australia; 4 School of Science, STEM College, RMIT University, Melbourne, Victoria, Australia; 5 German Diabetes Centre, Institute for Clinical Diabetology, Research Group Diabetic Nephropathy, Heinrich Heine University, Duesseldorf, Germany; 6 Department of Medicine and Therapeutics, The Chinese University of Hong Kong (CUHK), Hong Kong SAR, China; 7 Hong Kong Institute of Diabetes and Obesity, The Chinese University of Hong Kong (CUHK), Hong Kong SAR, China; 8 Li Ka Shing Institute of Health Sciences, The Chinese University of Hong Kong (CUHK), Hong Kong SAR, China; 9 School of Biomedical Sciences, The Chinese University of Hong Kong (CUHK), Hong Kong SAR, China; 10 Department of Biomedical Sciences, City University of Hong Kong, Hong Kong SAR, China; 11 University College Copenhagen, Faculty of Health, Department of Technology, Biomedical Laboratory Science, Copenhagen, Denmark

**Keywords:** diabetes, diabetes mellitus, diabetic nephropathy, endothelium, gene expression, gene transcription, diabetic kidney disease

## Abstract

**Key Points:**

- Set7 knockout improves diabetic glomerular structure and function and prevents diabetes-induced endothelial–mesenchymal transition (EDMT) by regulating Igfbp5.
- Set7 knockdown prevents, and (R)-PFI-2 hydrochloride reverses, diabetes-induced EDMT by regulating insulin growth factor binding protein 5.
- Set7 regulates the phenotypic EDMT switch, and inhibiting the methyltransferase attenuates glomerular injury in diabetic kidney disease.

**Background:**

Hyperglycemia influences the development of glomerular endothelial cell damage, and nowhere is this more evident than in the progression of diabetic kidney disease (DKD). While the Set7 lysine methyltransferase is a known hyperglycemic sensor, its role in endothelial cell function in the context of DKD remains poorly understood.

**Methods:**

Single-cell transcriptomics was used to investigate Set7 regulation in a mouse model of DKD, followed by validation of findings using pharmacological and short hairpin RNA inhibition inhibition of Set7.

**Results:**

Set7 knockout (Set7KO) improved glomerular structure and albuminuria in a mouse model of diabetes. Analysis of single-cell RNA-sequencing data showed dynamic transcriptional changes in diabetic renal cells. Set7KO controls phenotype switching of glomerular endothelial cell populations by transcriptional regulation of the insulin growth factor binding protein 5 (IGFBP5). Chromatin immunoprecipitation assays confirmed that the expression of the *IGFBP5* gene was associated with mono- and dimethylation of histone H3 lysine 4 (H3K4me1/2). This generalizability was investigated in human kidney and circulating hyperglycemic cells exposed to TGF*β*1. We showed that the highly selective Set7 inhibitor (R)-PFI-2 hydrochloride attenuated indices associated with renal cell damage and mesenchymal transition, specifically (*1*) reactive oxygen species production, (*2*) *IGFBP5* gene regulation, and (*3*) expression of mesenchymal markers. Furthermore, renal benefit observed in Set7KO diabetic mice closely corresponded in human glomerular endothelial cells with (R)-PFI-2 hydrochloride inhibition or Set7 short hairpin RNA silencing.

**Conclusions:**

Set7 regulates the phenotypic endothelial–mesenchymal transition switch and suggests that targeting the lysine methyltransferase could protect glomerular cell injury in DKD.

**Podcast:**

This article contains a podcast at https://dts.podtrac.com/redirect.mp3/www.asn-online.org/media/podcast/JASN/2024_04_25_ASN0000000000000345.mp3

## Visual Abstract

**Figure:**
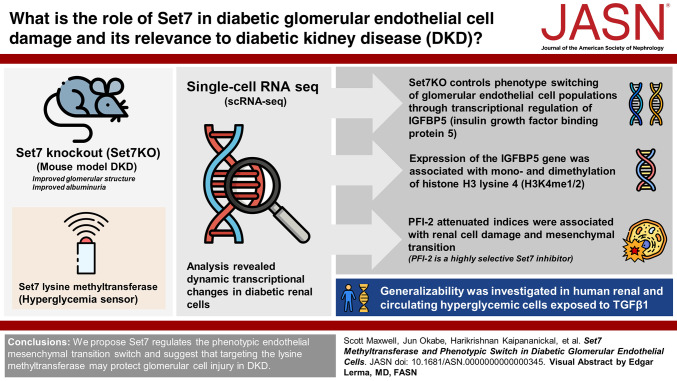

## Introduction

Diabetic kidney disease (DKD) is the leading cause of kidney failure,^1,2^ and chronic hyperglycemia is an important contributing factor to glomerular endothelial cell injury.^3^ Diabetes-induced glomeruli lesions can lead to mesangial expansion with podocyte loss and glomerular endothelial cell damage that influence hemodynamic indices early in the progression of DKD.^4,5^ In addition to maintaining glomerular homeostasis, endothelial cells undergo a process of mesenchymal transition (endothelial–mesenchymal transition or EDMT) that is a distinguishing event in the development of diabetic kidney fibrosis.^6,7^ While extracellular stimuli such as TGF*β*1, inflammatory molecules, and metabolic dysfunction can promote the transition,^8,9^ the mechanism of glomerular EDMT remains poorly understood in DKD.

The Set7 lysine methyltransferase is a metabolite-sensitive hyperglycemic sensor that is associated with proinflammatory gene expression.^10–12^ High-glucose conditions mediate Set7 signaling by writing mono- and dimethylation marks on histone H3 lysine 4 (H3K4me1/2).^13,14^ Set7 also regulates nuclear factor kappa-light-chain-enhancer of activated B cells activation activation, the expression of proinflammatory markers, and oxidative stress pathways in people with atherosclerotic vascular disease and type 2 diabetes mellitus.^15^ The effectiveness of Set7 to regulate profibrotic pathways relies on its methyltransferase capacity to tightly control lysine methylation of histone^16^ and nonhistone proteins.^17,18^ Recent studies have also shown that pharmacological Set7 inhibition attenuates renal fibrosis and kidney damage in rodent models.^19–21^ Indeed, Set7 regulates profibrotic signaling by TGF*β*1 activation under hyperglycemic conditions.

This article characterizes the hyperglycemic sensor Set7 with its role in glomerular endothelial cell function and diabetic kidney injury.

## Methods

Further details regarding the materials and procedures, including Supplemental Tables 1 and 2, are provided in the Supplemental Material.

### Diabetic Mouse Model

The Set7 deletion (Set7^−/−^, deletion of the functional membrane occupation and recognition nexus domain in exon 2) mice were generated by genOway (Lyon, France). The Set7^−/−^ mice were crossed with the ApoE^−/−^ mouse strain to generate double knockout animals (Set7^−/−^ApoE^−/−^). Six-week-old male Set7^+/+^ApoE^−/−^ and Set7^−/−^ApoE^−/−^ mice were rendered diabetic by five daily intraperitoneal injections of streptozocin (Sigma-Aldrich) at a dose of 55 mg/kg. At 10 weeks after the induction of diabetes, the levels of blood glucose and glycated hemoglobin (hemoglobin A1c) including other metabolic parameters were measured to confirm progression of diabetes. At 10 weeks, the animals were killed by CO_2_ overload. The kidneys were rapidly dissected and processed for subsequent analyses. All animal experiments were approved by the Alfred Medical Research and Education Precinct Animal Ethics Committee.

### Single-Cell RNA Sequencing

Preparation of single-cell suspension from fresh mouse kidney tissue was performed as described in the Online Data Supplement. Single-cell capture and transcriptomic profiling were performed using a droplet-based (Drop-Seq) technology and single-cell microfluidics platform (Dolomite Bio). Barcoded libraries were generated using the Nextera XT DNA Library Preparation Kit (Illumina) in house and sequenced by Novogene (Singapore). Single-cell data were processed and quantified with Drop-seq tools version 2.1 and further analyses using the R package Seurat version 3.^22,23^

### Cell Culture

Immortalized human glomerular endothelial cells were cultured and differentiated as previously described.^24^ For target validation experiment using the Set7 inhibitor, human immortalized cells were cultured with or without 15 *μ*M (R)-PFI-2 hydrochloride (PFI-2) (PFI-2; Cayman Chemicals) dissolved in DMSO for 24 hours before exposing them to 5.5 or 25 mM D-glucose in the presence or absence of 5 ng/ml TGF-*β*1 (R&D Systems) for 48 hours at 37°C.

### RNA and Protein Analysis

RNA and protein analyses were performed as described previously.^12^ Total RNA from the cells was extracted using TRIzol (Life Technologies) and the Direct-zol RNA Mini prep kit (Zymo Research) according to the manufacturer's instructions. cDNA synthesis and quantitative real-time reverse-transcription PCR analysis of gene expression were performed as previously described.^12,25,26^ Real-time quantitative PCR was performed by ABI Prism 7500 using the primers shown in Supplemental Table 1. Whole-cell extract preparation and histone extraction were performed as described previously.^12^ Histone methyltransferase activity assay was performed as described previously.^12,25,26^ Protein blotting signals were quantified by an infrared imaging system (Odyssey; LI-COR). An expanded description and the other assays are provided in the Data Supplement.

### Chromatin Immunoprecipitation

Chromatin immunoprecipitation assay was performed as described previously ^11,12^ using anti-H3K4me1 (Active Motif) and anti-H3K4me2 (Millipore) antibodies. The promoter regions of the human insulin growth factor binding protein 5 (IGFBP5) gene were amplified by quantitative real-time PCR, and the primers are provided in Supplemental Table 2.

### Statistical and Bioinformatic Analyses

Data are represented as mean±SEM. Statistical significance was determined using Student t-tests and one- or two-way ANOVA with Tukey *post hoc* test for multiple comparisons as necessary in Prism 9 (Graphpad). A *P* value < 0.05 was considered statistically significant.

### Data Availability

All raw sequencing data are available in NCBI's Gene Expression Omnibus database GSE158626.

## Results

### Set7KO Improved Diabetic Glomerular Structure and Function

**Figure 1:**
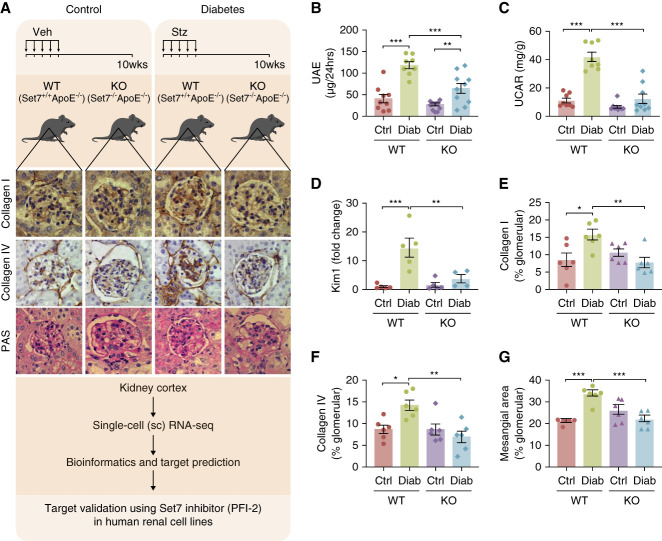
**Set7KO improves glomerular structure and function in a model of diabetes**. (A) Experimental design. Diabetes was induced in Set7WT (Set7^+/+^ApoE^−/−^) and Set7KO (Set7^−/−^ApoE^−/−^) mice using STZ. The kidney cortex was isolated after 10 weeks of diabetes and assessed by histological staining. Comparative scRNA-seq of the cortex identified Set7-dependent genes, and target validation was performed in human cell lines. Increased (B) UAE, (C) UCAR, (D) Kim1 expression, (E) glomerular collagen I and (F) collagen IV, and (G) mesangial area expansion in DKD were reduced by Set7KO. Mesangial area was determined by PAS staining. Data represented as mean±SEM. *P < 0.05, **P < 0.01, ***P < 0.001. *n*≥6 per group. Ctrl, control; Diab, diabetes; DKD, diabetic kidney disease; Kim1, kidney injury molecule 1; KO, knockout; PAS, periodic acid–Schiff; PFI-2, (R)-PFI-2 hydrochloride; scRNA-seq, single-cell RNA-sequencing; STZ, streptozocin; UAE, urinary albumin excretion; UCAR, urine albumin–creatinine ratio; Veh, vehicle; WT, wild type.

To assess the role of Set7 in DKD development, we generated the Set7-deficient (Set7^−/−^) mouse by the deletion of exon 2 of the *Setd7* gene. The Set7^−/−^ mice were crossed with apolipoprotein E–deficient (ApoE^−/−^) mice to accelerate DKD and vascular complication.^27^ We confirmed that Set7 and ApoE were absent at the mRNA level in the kidney cortex isolated from Set7^−/−^ApoE^−/−^ (referred to as Set7KO) mice (Supplemental Figure 1). To induce diabetes, streptozocin was administered over 5 consecutive days in the Set7KO mice. At 10 weeks, renal parameters were monitored, including the regulatory effect of Set7KO on the transcriptome using single-cell RNA-sequencing (scRNA-seq). Furthermore, the pharmacological Set7 inhibitor PFI-2^28^ was used for gene target validation in human kidney cells (Figure 1A). We observed an increase in blood glucose and glycated hemoglobin (hemoglobin A1c) and decreased body weights of diabetic mice when compared with nondiabetic controls (Table 1). In contrast to the diabetic animals, water intake and urine output were improved in Set7KO mice. Kidney parameters, including elevated urinary albumin excretion and albumin–creatinine ratio (Figure 1, B and C), kidney injury molecule 1 expression in the kidney cortex (Figure 1D), glomerular collagens I and IV (Figure 1, E and F), and mesangial area expansion (Figure 1G), were similarly improved in the diabetic Set7KO mice.

**Table 1:** General physiological parameters in Set7WT and Set7KO mice at 10 weeks of diabetic induction

Parameter	Set7WT		Set7KO	
	Control	Diabetes	Control	Diabetes
Blood glucose, mg/dl	217.1±9.3	553.5±15.9^a^	210.9±7.3	498.6±24.5^a^
Glycated hemoglobin, %	4.3±0.11	11.4±0.61^a^	4.2±0.08	10.5±0.43^a^
Body weight, g	31.0±0.61	24.4±0.59^a^	29.4±0.44	25.5±0.63^a^
Kidney wt/body wt, %	0.75±0.02	0.92±0.03^b^	0.75±0.03	0.90±0.06^b^
Food intake, g/24 h	3.1±0.13	4.9±0.17^a^	2.8±0.18	4.0±0.38^c^^,^^d^
Water intake, ml/24 h	3.3±0.52	18.8±1.23^a^	2.7±0.33	13.6±1.91^a^^,^^e^
Urine output, ml/24 h	0.9±0.11	14.6±1.39^a^	1.0±0.11	9.8±1.51^a^^,^^e^

*n*≤9 per group. Data are the mean±SEM. Kidney wt/body wt (%), kidney weight/body weight (%); KO, knockout; WT, wild type.

a *P* < 0.001 versus respective control.

b *P* < 0.05 versus respective control.

c *P* < 0.01 versus respective control.

d *P* < 0.05 versus diabetic Set7WT.

e *P* < 0.01 versus diabetic Set7WT.

### Set7 Regulates Pathways Associated with Diabetic Kidney Injury

**Figure 2:**
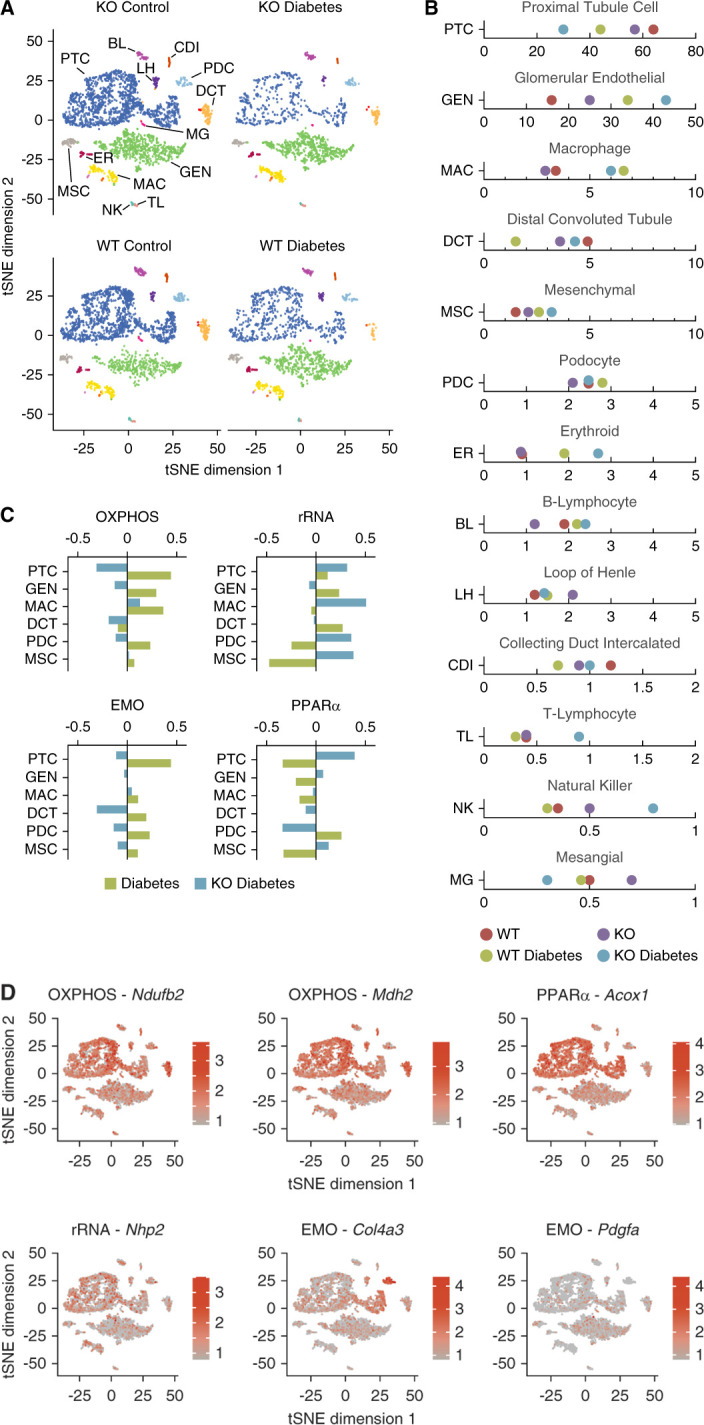
**Influence of Set7 deletion in diabetic kidney transcriptome revealed by scRNA-seq**. (A) scRNA-seq cell clusters in the diabetic kidney were visualized by t-distributed stochastic neighbor embedding (tSNE) after analysis using Seurat and R package. (B) Composition of the cell clusters in each experimental group. Significant changes in cell composition between experimental groups (ANOVA) included the proximal tubule (*P* < 0.05) and GEN (*P* = 0.066) clusters, as calculated using the *propeller* method. (C) GSEA identifies four reactome pathways that belong to OXPHOS, rRNA processing, EMO, and PPAR*α* activation. These pathways were significantly changed by Set7 in diabetes. (D) Distribution of diabetic Set7-dependent gene expression was visualized by tSNE. Color gradient indicates the number of transcripts detected per cell. BL, B-lymphocyte; CDI, collecting duct intercalated; DCT, distal convoluted tubule; EMO, extracellular matrix organization; ER, erythroid; GEN, glomerular endothelial; GSEA, gene set enrichment analysis; LH, loop of Henle; MAC, macrophage; MG, mesangial; MSC, mesenchymal; NK, natural killer; OXPHOS, respiratory electron transport; PDC, podocyte; PPARα, peroxisome proliferator-activated receptor alpha; PTC, proximal tubule cell; rRNA, ribosomal RNA; TL, T-lymphocyte.

To identify the complete transcriptomic changes regulated by Set7, we performed high-depth scRNA-seq in diabetic groups. Analysis identified proximal tubule cells, glomerular endothelial cells, macrophages, mesenchymal cells, and podocytes as the major cell types in the kidney cortex (Figure 2A and Supplemental Figure 2). While the number of proximal tubule cells were reduced in the diabetic group (*P* = 0.05), we observed an increase in the glomerular endothelial cell cluster (*P* = 0.07) in the diabetic Set7KO group (Figure 2B). To assess diabetes-associated pathways regulated by Set7, we performed gene set enrichment analysis (GSEA) on the scRNA-seq clusters and identified four major reactome pathways involving respiratory electron transport (abbreviated as OXPHOS), ribosomal RNA (rRNA) processing, extracellular matrix organization (EMO), and peroxisome proliferator-activated receptor alpha (PPAR*α*) activation (Figure 2C). Representative genes from these pathways were assessed for expression, specifically *Ndufb2* and *Mdh2* (OXPHOS), *Acox1* (PPAR*α* activation), *Nhp2* (rRNA processing), and the gene group comprising *Col4a3* and *Pdgfa* (EMO). *Ndufb2*, *Mdh2*, and *Acox1* were highly expressed in proximal tubule cells (Figure 2D). We observed the expression of *Col4a3* was highest in podocytes, whereas *Pdgfa* was distributed between the glomerular endothelial and tubule cell clusters. These single-cell data suggest that Set7KO may regulate major pathways that are associated with diabetic kidney injury.

### Set7 Controls the Glomerular Endothelial Transcriptome in DKD

**Figure 3:**
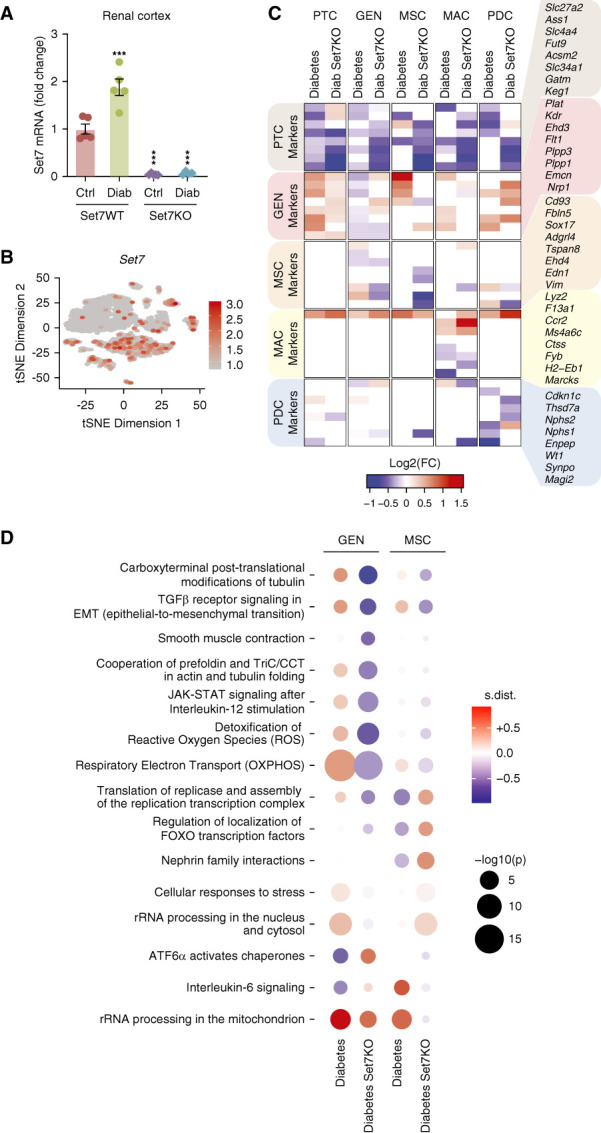
**Set7 regulates the GEN transcriptome**. (A) Increased Set7 expression in the kidney cortex derived from diabetic mice. Expression of Set7 mRNA in the kidney cortex was assessed by qRT-PCR. *n*=5 per group. Data are represented as mean±SEM. ***P < 0.001 versus control Set7WT mice. (B) Cell distribution of Set7 expression in the kidney cortex by scRNA-seq. (C) Set7KO regulates the expression of GEN and mesenchymal markers in the diabetic kidney. Color gradient indicates significant (*P* < 0.05) log fold changes for each gene: red (elevated), blue (reduced), and white (no significant change; *P* > 0.05). (D) The top 15 reactome pathways that are significantly changed by diabetes (control versus diabetic Set7WT) and Set7 deletion in diabetes (Diab Set7KO; diabetic Set7WT versus diabetic Set7KO) are illustrated in GEN and mesenchymal cell clusters using GSEA. qRT-PCR, quantitative real-time reverse-transcription PCR.

Single-cell RNA-seq showed dynamic transcriptional regulation by Set7 in the kidney cortex. Consistent with previous studies,^20,29^ we observed increased Set7 mRNA expression in the diabetic kidney (Figure 3A) consequentially in the podocytes (24%), mesenchymal (MSC, 10%), and glomerular endothelial cell (8%) clusters that were barely detectable in proximal tubule cells (0.8%) (Figure 3B). Despite the increase in glomerular endothelial cell numbers (shown previously in Figure 2B), Set7KO reduced the expression of genes that define glomerular endothelial cell identity in diabetic animals (Figure 3C). Indeed, we observed segregation of the glomerular endothelial cell population from the cell cluster defining mesenchymal identity with reduced gene expression in the diabetic Set7KO mice. This influence on the transcriptome was consistent with a phenotypic switch involving the glomerular endothelial cell population. Gene set enrichment analysis of the glomerular endothelial and mesenchymal clusters found the principal pathway regulating epithelial-to-mesenchymal transition (EMT) was inversely correlated: while the EMT pathway was upregulated in the diabetic group, EMT was downregulated by Set7KO in diabetic mice (Figure 3D). These results suggest that Set7 is closely associated with diabetic glomerular endothelial-to-mesenchymal transition.

**Figure 4:**
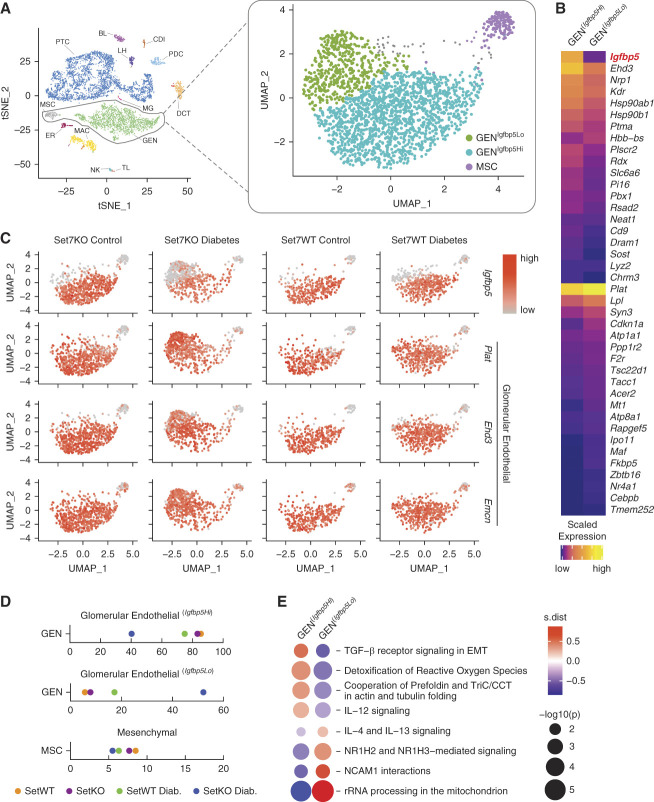
**Set7-dependent regulation of the GEN transcriptome in diabetic mouse kidney**. (A) Identification of the novel GEN cell population in diabetic Set7KO mouse kidney. Reclustering of the GEN and mesenchymal populations in our scRNA-seq data. (B) Top 40 genes significantly changed in the GEN (*Igfbp5 high*) and GEN (*Igfbp5 low*) clusters. (C) Distribution of *Igfbp5* and *Plat* gene markers in each experimental group is illustrated. (D) Composition of the cell clusters in each experimental group. Significant changes in cell composition between experimental groups (ANOVA) included GEN (*Igfbp5 high*, *P* < 0.05) and GEN (*Igfbp5 low*, *P* < 0.01) and calculated using the *propeller* method. (E) Reactome pathway exchanges observed in the GEN (*Igfbp5 high*) and GEN (*Igfbp5 low*) cell clusters. EMT, epithelial-to-mesenchymal transition; IGFBP5, insulin growth factor binding protein 5; UMAP, uniform manifold projection.

Unsupervised clustering of cell populations (Figure 4A) found two glomerular endothelial subpopulations on the basis of *Igfbp5* expression levels, designated as glomerular endothelial (*Igfbp5 high*) and glomerular endothelial (*Igfbp5 low*) (Figure 4, B and C). The regulatory dosage of renal *Igfbp5* is critical because the transcription factor is a marker of endothelial identity^30,31^ and is closely associated with the development of DKD.^32,33^ While *Igfbp5* expression was elevated in diabetic Set7WT mice, mRNA levels remain unchanged in the kidney cortex in Set7KO mice (Supplemental Figure 3A). Functionally divergent, *Igfbp5* was predominantly expressed in the glomerular endothelial cell population (Supplemental Figure 3B). While the glomerular endothelial (*Igfbp5 high*) cells account for more than 75% of the endothelial population in diabetic mice, this is significantly reduced (*P* = 0.03) in the diabetic Set7KO group (Figure 4D). By contrast, the glomerular endothelial (*Igfbp5 low*) cell cluster (*P* = 0.01) was remarkably elevated in the kidney cortex of diabetic Set7KO mice (Figure 4, C and D).

Whereas glomerular endothelial *Igfbp5* high and low cells clustered separately from their respective control groups, scRNA-seq confirmed glomerular endothelial cell identity with strong expression of *Plat* (plasminogen activator tissue type), *Ehd3* (Eps15 homology domain-containing protein 3), and *Emcn* (endomucin) in glomerular endothelial (*Igfbp5 high*) and glomerular endothelial (*Igfbp5 low*) groups. Importantly, GSEA identified pathways involved with EMT, and reactive oxygen species (ROS) were upregulated in the glomerular endothelial (*Igfbp5 high*) cell population but downregulated in the glomerular endothelial (*Igfbp5 low*) cell cluster (Figure 4E). These results suggest that Set7 regulates dosage of *Igfbp5* expression in the glomerular endothelial transcriptome influencing DKD.

### PFI-2 Prevented Expression of Diabetes-Associated Renal Genes

**Figure 5:**
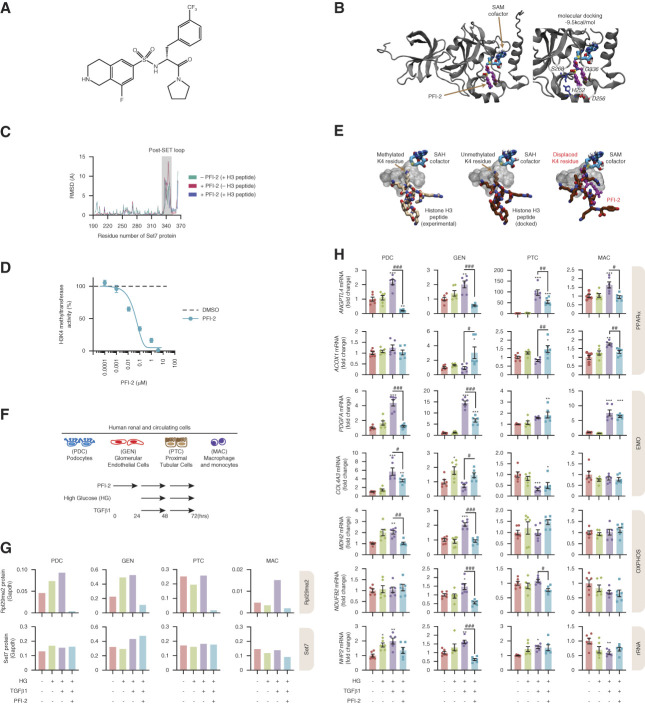
**PFI-2 attenuates diabetes-related pathways and genes in human renal cells**. (A) Chemical structure of the SET7 inhibitor PFI-2. (B) Molecular docking was used to evaluate the binding characteristics of PFI-2 within the substrate peptide-binding groove of the SET7 protein. The binding affinity (kcal/mol) and key residues are shown. The italicized residues were predicted to form hydrogen bonds with PFI-2. Polar residues are in dark blue, while negatively charged residues are in red. (C) The per-residue RMSD (Å) was calculated and the conformational changes that occur in the post-SET loop (shown in shade). (D) Inhibition of Set7 histone methyltransferase activity by PFI-2. FLAG tagged Set7 protein (FLAGSet7) was incubated with [H^3^]-S-adenosyl-methionine and histone H3 peptide in the presence of PFI-2. Tritiated histone H3 peptide was measured by liquid scintillation. Activity is presented as percentage, 100% corresponding to FLAGSet7 with DMSO vehicle control. Data are presented as mean±SEM, *n*=3. (E) Protein-peptide docking was performed using the HPEPDOCK server. The histone H3 peptide was modified to contain an unmethylated lysine residue (K4) and was docked to structures of SET7 in the absence (left and middle) and presence of PFI-2 (right) within the peptide-binding groove. (F) Schematic of the experimental design to assess the Set7 inhibitor PFI-2 in human cell lines under diabetic conditions. Cells were treated with PFI-2 for 24 hours before hyperglycemic stimulation with TGF*β*1 for 48 hours. (G) Set7 inhibition by PFI-2 in human cell lines. Demethylation of Rpl29 and Set7 levels were quantified by Li-COR Odyssey. Gapdh was used as loading control. (H) PFI-2 attenuates the expression of TGF*β*1-induced genes in human cell types. The expression of genes shown in brackets belong to the four core pathways: OXPHOS (*MDH2*, *NDUFB2*), PPAR*α* (*ACOX1*, *ANGPTL4*), rRNA (*NHP2*), and EMO (*PDGFA*, *COL4A3*). Gene expression was assessed by qRT-PCR. The assessment of *ANGPTL4* expression was used as a TGF*β*1 response gene. *n*=6 per group. Data are represented as mean±SEM. *P < 0.05, **P < 0.01, ***P < 0.001 versus normal glucose control; #*P* < 0.05, ##*P* < 0.01, ###*P* < 0.001 versus diabetic condition (HG+TGF*β*1). HG, high glucose; RMSD, root-mean-square deviation; SAH, *S*-adenosyl homocysteine; SAM, *S*-adenosyl methionine.

To assess whether inhibition of the hyperglycemic sensor Set7 could prevent EDMT and was consistent with a model in which histone methylation is coupled with transcriptional output, we stimulated clinically relevant human cells with PFI-2^28^ as shown in Figure 5A. To assess the conformational changes in the C-terminal domain upon substrate binding, the crystal structure of SET7 in complex with *S*-adenosyl methionine or *S*-adenosyl homocysteine, the histone H3 peptide and PFI-2 were compared. Molecular docking analysis indicated PFI-2 bound to the substrate peptide-binding groove with an affinity of −9.5 kcal/mol (Figure 5B). PFI-2 was predicted to form hydrogen bonds with G336 and the polar residue S268. On the basis of the per-residue root-mean-square deviation analysis, the post-SET loop (G336-A349) displayed the highest degree of variability (Figure 5C). We confirmed specific inhibition of H3K4 methyltransferase activity by PFI-2 (Figure 5D). To investigate the binding mode of the unmethylated histone H3 peptide to SET7 in the absence and presence of PFI-2, protein–peptide docking was performed using the HPEPDOCK algorithm. In the absence of PFI-2, the unmethylated histone H3 peptide was found to preferentially bind to the substrate peptide-binding groove and the orientation coincided with the experimentally determined structure of the methylated histone H3 peptide (Figure 5E). Although the unmethylated histone H3 peptide maintained its preference for the peptide-binding groove in the presence of PFI-2, the target lysine residue (K4) was displaced from the lysine-binding channel (Figure 5E, right). Because hyperglycemia activates the TGF*β* response^34,35^ and we demonstrated that Set7KO regulates key renal cell clusters, we stimulated human podocytes, glomerular endothelial cells, macrophage monocytes, and proximal tubular cells with PFI-2 before high-glucose and TGF*β*1 stimulation (Figure 5F). We confirmed that PFI-2 inhibited methyltransferase activity in these cells by quantitative analysis of the ribosomal Rpl29me2 protein,^36^ a reliable biomarker of Set7 activity (Figure 5G). Next, we assessed the expression of genes identified by scRNA-seq in diabetic Set7KO mice (as shown previously in Figure 2D). PFI-2 attenuated the activation of *ANGPTL4*, which is involved in TGF*β*1-dependent glucose homeostasis^37^ (Figure 5H). Moreover, PFI-2 enhanced expression of the fatty-acid oxidation target gene *ACOX1* in the glomerular endothelial cell and proximal tubule cell clusters. These results closely correspond with the transcriptome changes observed in the Set7KO mice. Because diabetic fibrosis is also characterized by excessive deposition of extracellular matrix proteins,^2^ we show the activation of *PDGFA* was attenuated by PFI-2 in human podocyte and glomerular endothelial cells. Furthermore, PFI-2 reduced *COL4A3* mRNA levels in podocytes despite elevated expression in human glomerular endothelial cells. The activation of the core genes associated with the OXPHOS and rRNA processing pathways—*NDUFB2*, *MDH2*, and *NHP2*—were also attenuated by PFI-2 in glomerular endothelial cells. These results suggest that pharmacological Set7 inhibition in human cells closely corresponded with the expression of genes implicated in the four major pathways in the kidney cortex of diabetic mice.

### Set7 Regulation of IGFBP5

**Figure 6:**
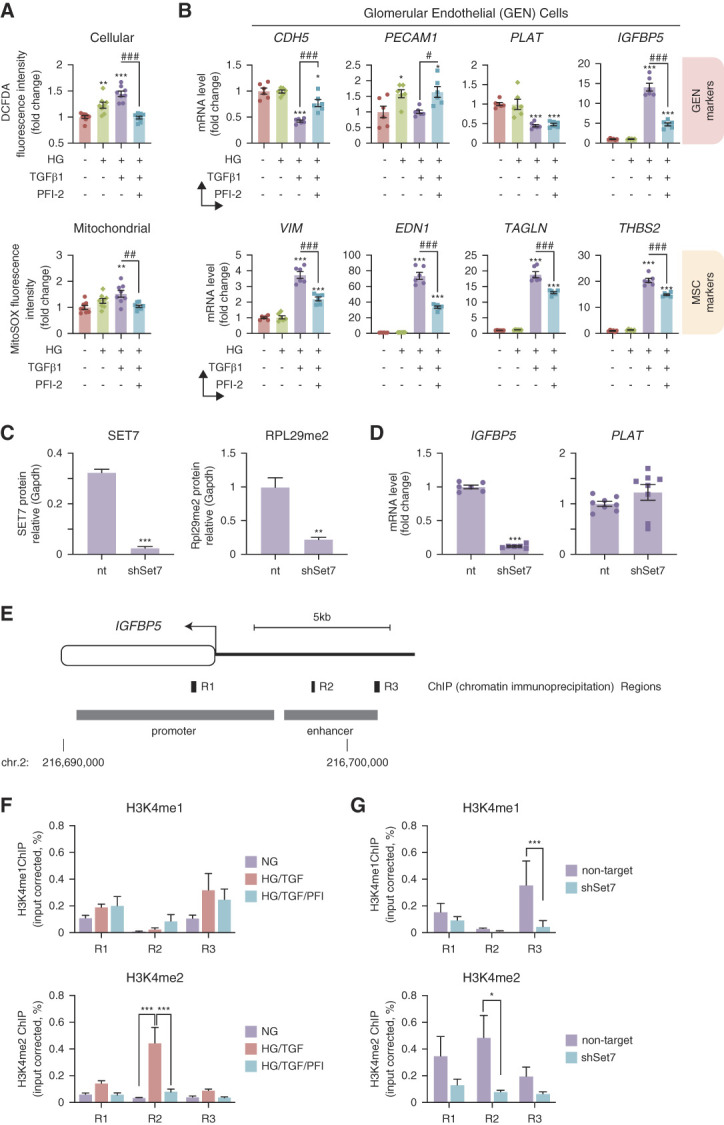
**Set7-dependent transcriptional regulation of IGFBP5 in human GEN cells**. (A) PFI-2 attenuates TGF*β*1-induced ROS levels in GEN cells. Cellular and mitochondrial ROS production was measured by fluorescence intensity using the fluorogenic dyes DCFDA and MitoSOX Red (Mitochondrial Superoxide Indicator). *n*=8 per group. (B) PFI-2 diminishes TGF*β*1-induced EDMT in hyperglycemic GEN cells. The expression of the endothelial (*CDH5*, *PECAM1*, *PLAT*, and *IGFBP5*) and mesenchymal markers (*VIM*, *EDN1*, *TAGLN*, and *THBS2*) were assessed by qRT-PCR. *n*=6 per group. (C) Set7 inhibition by shRNA in human GEN cells. Whole-cell extracts prepared from controls (non-target vector) or Set7 knockdown (shSet7) in GEN cells were analyzed by immunoblot. Rpl29 methylation and Set7 protein content were quantified by Li-COR Odyssey. Gapdh was used as loading control. *n*=3 per group. (D) Specific reduction of IGFBP5 in shSet7 GEN cells. *PLAT* and *IGFBP5* gene expression were assessed by qRT-PCR. *n*=6 per group. (E) Set7 regulates H3K4 methylation content of the human IGFBP5 gene. The regulatory promoter and enhancer elements derived from the GeneHancer database are illustrated. ChIP regions (R1, R2, and R3) were assessed for H3K4me1 and H3K4me2. (F and G) Soluble chromatin was fractionated from GEN cells and immunopurified with H3K4me1 or H3K4me2 antibodies. qPCR was used to assess DNA enrichment. (F) PFI-2 attenuates TGFβ1-induced H3K4 methylation at the IGFBP5 gene in human GEN cells. (G) shSet7 regulates H3K4 methylation in human GEN cells. *n*=3 per group. Normoglycemia (NG); hyperglycemia/TGF*β*1 (HG/TGF); hyperglycemia/TGF*β*1/PFI-2 (HG/TGF/PFI). Data are represented as mean±SEM. *P < 0.05, **P < 0.01, ***P < 0.001; #*P* < 0.05, ##*P* < 0.01, ###*P* < 0.001 versus diabetic condition (HG+TGF*β*1). ChIP, chromatin immunoprecipitation; DCFDA, 2′,7′-dichlorodihydrofluorescein diacetate; EDMT, endothelial–mesenchymal transition; qPCR, quantitative real-time PCR; ROS, reactive oxygen species; shRNA, short hairpin RNA.

Hyperglycemia-induced oxidative stress plays a critical role in the development of DKD. Hyperglycemic glomerular endothelial cells treated with PFI-2 attenuate TGF*β*1-induced cellular and mitochondrial ROS production (Figure 6A). If Set7 was responsible for EDMT in diabetic mice, then PFI-2 should prevent the expression of key EDMT genes. We stimulated human glomerular endothelial cells with TGF*β*1 to confirm reduced expression of the endothelial markers *CDH5* (cadherin 5), *PECAM* (platelet endothelial cell adhesion molecule), and *PLAT*, while upregulating the expression of mesenchymal gene markers *VIM* (vimentin), *EDN1* (endothelin-1), *TAGLN* (transgelin), and *THBS2* (thrombospondin-2) (Figure 6B). PFI-2 significantly improved the expression of EDMT genes in TGF*β*1-induced hyperglycemic glomerular endothelial cells. These results suggest that pharmacological Set7 inhibition may prevent the endothelial–mesenchymal phenotype switch. While we previously showed that *IGFBP5* activation in diabetic glomerular endothelial cells was attenuated by Set7KO in mouse (Figure 4D), we also show PFI2 prevented the expression of core EDMT genes in glomerular endothelial cells (Figure 6B). To validate Set7-mediated regulation of *IGFBP5* gene expression, we performed short hairpin RNA Set7 knockdown (shSet7) in human glomerular endothelial cells. We confirmed knockdown of Set7 protein was associated with methyltransferase activity by RPL29me2 (Figure 6C). Furthermore, shSet7 significantly reduced *IGFBP5* but not *PLAT* (Figure 6D). TGF*β*1 and high glucose did not influence *IGFBP5* expression in shSet7 glomerular endothelial cells (Supplemental Figure 4), suggesting that Set7 activity was required for *IGFBP5* expression. Because Set7 is known to methylate histone H3 lysine K4,^12,13^ we investigated mono- and di-methylation of H3K4 at the promoter region (R1) and enhancer regions (R2 and R3) of the *IGFBP5* gene directly by chromatin immunoprecipitation (Figure 6E). We demonstrated that elevated H3K4 methylation at the *IGFBP5* R2 site was significantly reduced by PFI-2 in hyperglycemic glomerular endothelial cells stimulated with TGF*β*1 (Figure 6F). Moreover, we showed reduced H3K4 methylation of the *IGFBP5* gene in shSet7 glomerular endothelial cells (Figure 6G). We also investigated chromatin accessibility at the *IGFBP5* promoter/enhancer region from publicly available single cell assay for transposase-accessible chromatin using sequencing (scATAC-seq) datasets for the human kidney (Supplemental Figure 5). The *IGFBP5* R2 site intersects with overlapping chromatin accessibility peaks between two independent scATAC-seq datasets generated from the human kidney. We observed altered chromatin accessibility in glomerular endothelium between control and CKD groups for the *IGFBP5* gene at R1 and R2 sites. Taken together, these results suggest that Set7 regulates H3K4 methylation at the enhancer regions of the *IGFBP5* gene in human glomerular endothelial cells.

### Preventing Diabetic Phenotypic Switch in Mouse and Human Glomerular Endothelial Cells

To understand the influence of Set7 on the transcriptome, we profiled human glomerular endothelial cells using the small molecule inhibitor PFI-2 or by the viral shSet7 knockdown using mRNA sequencing (Supplemental Figure 6A). Genes upregulated in expression by high glucose and TGF*β*1 were attenuated by targeting Set7 activity (Supplemental Figure 6B) involving the EMO and IGFBP signaling pathways (Supplemental Figure 6C). Consistent with our previous studies in diabetic mice, we confirm that *IGFBP5* expression was dependent on Set7-mediated regulation in glomerular endothelial cells.

**Figure 7:**
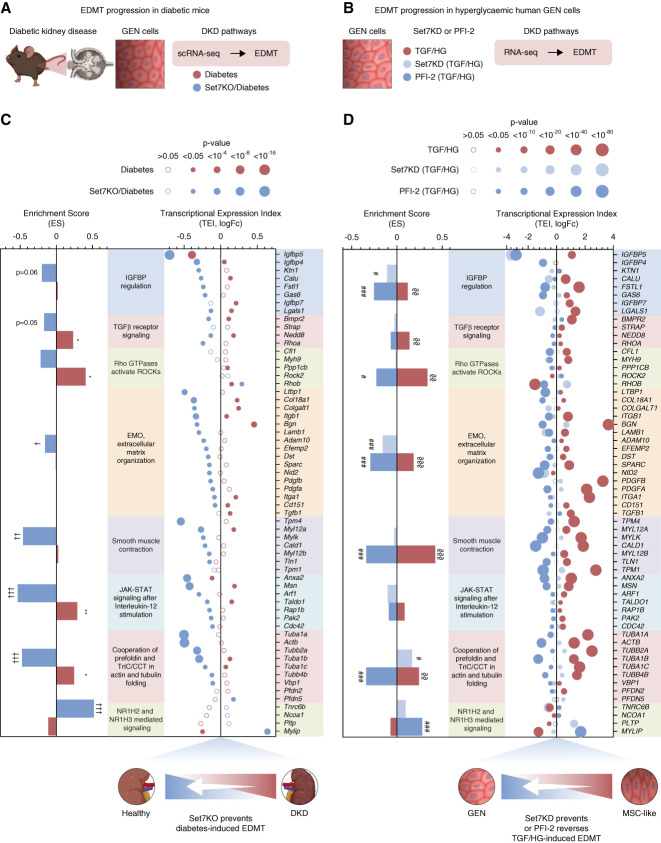
**Targeting Set7 in diabetic GEN transcriptome**. Set7-mediated endothelial transcriptomes in the GEN population derived from (A) diabetic mice and (B) human GEN cells stimulated with TGF*β*1 and HG (TGF/HG) conditions. (C) scRNA-seq analysis demonstrates the transcriptional expression index (TEI) of Set7-dependent diabetic pathways in diabetic mice include the following: IGFBPs regulation, TGF*β* receptor signaling, Rho GTPases-activated ROCKs, EMO, smooth muscle contraction, JAK-STAT signaling after IL-12 stimulation, cooperation of prefoldin and TriC/CCT in actin and tubulin folding, and NR1H2- and NR1H3-mediated signaling pathways are identified. *P < 0.05, **P < 0.01 versus nondiabetic mice control; †*P* ≤ 0.05, ††*P* < 0.01, †††*P* < 0.001 versus diabetic Set7WT mice. (D) Influence of Set7 inhibition by PFI-2 or shRNA in human GEN cells identifies Set7-dependent diabetic genes and pathways. Genes were filtered by *P* value < 0.05 in either TGF/HG or Set7 inhibition in TGF/HG; §§*P* < 0.01, §§§*P* < 0.001 versus nondiabetic condition (NG); #*P* < 0.05, ##*P* < 0.01, ###*P* < 0.001 versus diabetic (TGF/HG) condition.

While previous studies have suggested that Set7 inhibition can improve renal fibrosis,^19–21,38^ the regulatory pathways involved in EDMT remain poorly understood. To study the generality that the Set7 methyltransferase could regulate the phenotype switch, we compared the transcriptome changes in mouse and human glomerular endothelial cells (Figure 7, A and C). We first examined pathways that were directly regulated by diabetes in glomerular endothelial cells and reversed by Set7KO in mice. Transcriptome changes assessed by scRNA-seq identified the regulation of the *IGFBP* pathway, including TGF*β* receptor signaling, extracellular matrix organization (EMO), smooth muscle contraction, and IL-12 signaling. Enrichment scores for pathways regulated by diabetes and prevented by Set7KO, including statistical significance, are shown in the left panel of Figure 7B. RNA-seq shows the transcriptional expression index (TEI) of genes shift toward recovery in Set7KD diabetic mice by preventing EDMT. Endothelial Set7 is a critical hyperglycemic sensor that regulates gene expression in primary human vascular cells. To determine whether the phenotypic switch observed in diabetic mice was consistent with a model in which Set7 was coupled with IGFBP5 control, we assessed the transcriptome of hyperglycemic human glomerular endothelial cells stimulated by TGF (Figure 7C). We observed a parallel association of EDMT pathways in human Set7KD glomerular endothelial cells that was consistent with diabetic Set7KO mice preventing the phenotype switch (Figure 7D). RNA-seq revealed the core pathways associated with EDMT include JAK-STAT signaling, cytoskeletal organization (TriC/CCT), and NR1H2-mediated NR1H3 pathways.^7–9,39–41^ Consistent with these observations, we also report that pharmacological inhibition of Set7 by PFI-2 can regulate DKD pathways associated with the phenotype switch. These results suggested that targeting *IGFBP5* regulation by Set7 prevents diabetes-induced EDMT in mouse and human glomerular endothelial cells.

## Discussion

**Figure 8:**
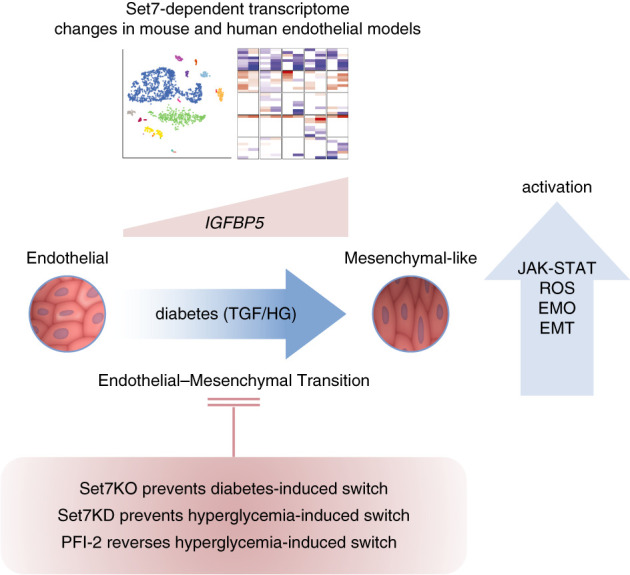
**Schematic model of Set7-mediated regulation of phenotypic switch**. In diabetic GEN cells, Set7 activates IGFBP5 expression by regulating H3K4me1 and H3K4me2. The mesenchymal-like cells are hypothesized to transition and activate ROS, EMO, JAK-STAT, and EMT pathways. Genetic knockout and pharmacological inhibition of Set7 attenuates ROS production and improves fibrosis and proinflammatory pathways and the expression of EDMT genes implicated in DKD.

In the context of DKD, hyperglycemia plays a significant role in the progression of EDMT; however, the molecular mechanisms that regulate this phenotype switch remain poorly understood. The role of the glucose sensor Set7 is largely unknown. We have combined mouse genetics, single-cell transcriptome, and biochemical approaches to show that the Set7 methyltransferase advances the phenotypic switch in diabetic kidney injury. Renal fibrosis is a pathological feature of excessive deposition of extracellular matrix in the diabetic kidney, and our studies show that Set7 regulates the expression of genes critical to fibrosis, including endothelial barrier function and mitochondrial ROS production (Figure 8). Glomerular endothelial cells express the highest Set7 levels, and this corresponds with EDMT that is implicated in microalbuminuria generation in DKD.^7^ Our studies also show that the mesenchymal gene markers upregulated by diabetes in mice were attenuated in human glomerular endothelial cells by PFI-2.

These results are novel for several reasons. First, albuminuria and glomerular structure were dramatically improved in the diabetic Set7KO mice. While albuminuria is a clinical manifestation of glomerular filtration barrier destruction,^5^ the molecular mechanisms that drive this process in diabetic kidney injury remain poorly defined. Our studies show that Set7 is predominantly expressed in podocyte and glomerular endothelial cell types that are considered to be functionally important in glomerular filtration. Furthermore, scRNA-seq analyses revealed the glomerular endothelial cell populations were dynamically altered in the diabetic Set7KO kidney cortex. Nowhere is this more evident than the effect on core pathways, such as extracellular matrix organization, IL-12 signaling, and TGF*β* receptor signaling observed in mouse and human glomerular endothelial cells. Previous studies have shown a context-dependent role for vascular Set7 in proinflammatory gene activation,^10,11,13^ and the experimental results presented here suggest that diabetic kidney injury associated with hyperglycemia regulate endothelial inflammation and progressive damage of the glomerular filtration barrier.

A key challenge in endothelial biology is explaining how glomerular cells acquire distinct mesenchymal fates in response to hyperglycemic signaling cues. For example, extracellular glucose does not carry intracellular instructions that mediate epigenetic change unless there are methyltransferase sensors that perceive hyperglycemia to regulate gradient-dependent gene expression. Endothelial Set7 is a glucose-dependent sensor that regulates histone code changes and the activation of vascular gene expression in response to transient and chronic hyperglycemia.^12^ Furthermore, hyperglycemia promotes nuclear Set7 localization to activate proinflammatory gene expression that is distinguished by histone methylation. This could explain the nucleosomal H3K4 preference we observe of Set7 methylation associated with IGFBP5 chromatin to directly regulate gene expression in response to hyperglycemia. In this study, we show Set7 was correlated in mouse and human cell studies using genetic and pharmacological approaches that attenuated the mesenchymal markers of expression influencing ROS overproduction. This is consistent with the role of Set7 in mesenchymal differentiation^25^ and does not exclude the immunometabolic changes associated with trained immunity.^36^ Set7 enzyme methylates not only lysine 4 residue of histone H3, which is implicated in gene-activating transcriptional events, but can also methylate lysine residues of nonhistone proteins. For example, Set7 can regulate TGF*β*1-mediated extracellular matrix genes by methylation of histone H3, SMAD7, and SMAD3.^16–18^ We have previously demonstrated that Set7 controls the expression of mesenchymal markers by methylation of histone H3 and serum response factor transcription factor.^25^ These observations support a role for Set7 regulation of EDMT in glomerular endothelial cells in response to diabetic stimuli. Consistent with its role as a hyperglycemic sensor, we show that the expression of vascular cell proliferation and differentiation markers—*PDGFA*, *PDGFB*, and *BGN*^42,43^—were significantly reduced by PFI-2.

In diabetic glomerular endothelial cells, we have shown that *Igfbp5* is a primary Set7 gene target in mice using scRNA-seq analyses. IGFBP5 is an important secretory protein that is related to inflammation and fibrosis in DKD^44^ and a kidney-specific endothelial cell marker.^30,31,45^ While upregulation of IGFBP5 has been associated with DKD,^32,33,46^ the mechanism of regulation remains poorly understood. Recent studies have shown that IGFBP5 activates inflammation signaling directly by influencing endothelial glycolysis in diabetic mice.^47^ Furthermore, hyperglycemia induces the IGFBP5-mediated cellular processes in glomerular endothelial, mesangial, and myocardial cells.^47–49^ We have shown that Set7 plays an important role for *IGFBP5* human gene regulation mediated by H3K4me1/2 on the enhancer region in glomerular endothelial cells. Because IGFBP5 expression was not elevated by TGF*β*1 in Set7KD glomerular endothelial cells, we hypothesize that lysine methyltransferase is required for transcriptional expression of the gene. Furthermore, the IGFBP5 protein can translocate to the nucleus to exert its activity in an IGF-independent manner.^50^ The subcellular localization of Set7 is also induced by high glucose levels and associated with transcriptional changes of the inflammatory and extracellular matrix genes pathways.^12^ Because Set7KO reduced collagen IV expression in peritubular capillaries, it is plausible that Set7 could play a role in peritubular capillary endothelial cells in the diabetic setting. Future studies examining protein interaction and endothelial cell types may provide new insights of the phenotype switch in early development of diabetes-related complications.

The experimental results in this article suggest the dynamic transcriptional changes by PFI-2 attenuated EMO, IL-12 signaling, and EMT pathways, as well as IGFBP5 in human glomerular endothelial cells, closely correspond with the pathways improved by Set7KO in diabetic mice. While progress with single-cell technology has meant that the capacity to uncover the physiological targets in the kidney is now possible, a limitation of our preclinical studies is applied translation. Although PFI-2 is not licensed for diabetic complications, including DKD, the findings provide a proof of concept to translate these studies and the continued development of novel Set7 inhibitors.^51^ Taken together, our findings have provided new insights into Set7 regulating EDMT and IGFBP5. Our studies highlight the prospect of targeting Set7 lysine methyltransferase and the phenotype switch in diabetic kidney injury.

## Supplementary Material

## Data Availability

Sequencing data are available in NCBI's Gene Expression Omnibus (GEO) database GSE158626.

## Supplemental Material

Supplemental Table 1. Primer sequences used for quantitative RT-PCR assays.

Supplemental Table 2. Primer sequences used for ChIP assay.

Supplemental Figure 1. mRNA expression in the kidney cortex derived from Set7^−/−^ApoE^−/−^ mice.

Supplemental Figure 2. Violin plots of cluster marker genes identified using the Seurat function “FindAllMarkers.”

Supplemental Figure 3. *Igfbp5* mRNA expression patterns.

Supplemental Figure 4. Gene expression profiles in human shSet7 glomerular endothelial cells treated with high glucose (HG), TGF*β*1, and Set7 inhibitor (PFI-2).

Supplemental Figure 5. Chromatin accessibility at the IGFBP5 promoter and enhancer regions from publicly available scATAC-seq human kidney datasets.

Supplemental Figure 6. RNA-seq analysis of human glomerular endothelial cells treated with high glucose, TGF*β*1 (TGF/HG), and Set7 inhibitions (Set7KD or PFI-2).
