# Supplementary material for: Set7 Methyltransferase and Phenotypic Switch in Diabetic Glomerular Endothelial Cells
Source: J Am Soc Nephrol. 2024 Apr 17;35(6):733–48. doi: 10.1681/ASN.0000000000000345 (PMC11164123; doi:10.1681/ASN.0000000000000345)
Supplement: Supplementary file 1 [file jasn-35-733-s001.pdf]

ASN American Society of Nephrology | JASN Journal of the American Society of Nephrology | KIDNEY360 Accessing Our World From Every Angle | CJASN Clinical Journal of the American Society of Nephrology

ASN Journal Disclosure Form

As per ASN journal policy, I have disclosed any financial relationship or commitment held by myself and/or my spouse/partner in the past 36 months as included below. I have listed my Current Employer below to indicate there is a relationship requiring disclosure. If no relationship exists, my Current Employer is not listed.

K. Al-Hasani has nothing to disclose.

I understand that the information above will be published within the journal article, if accepted, and that failure to comply and/or to accurately and completely report the potential financial conflicts of interest could lead to the following: 1) Prior to publication, article rejection, or 2) Post-publication, sanctions ranging from, but not limited to, issuing a correction, reporting the inaccurate information to the authors' institution, banning authors from submitting work to ASN journals for varying lengths of time, and/or retraction of the published work.

Name: Keith Al-Hasani
Manuscript ID: ASN-2023-001601R1
Manuscript Title: Set7 Methyltransferase Regulates the Phenotypic Switch in Diabetic Glomerular Endothelial Cells
Date of Completion: March 6, 2024
Disclosure Updated Date: March 6, 2024

ASN American Society of Nephrology JASN Journal of the American Society of Nephrology KIDNEY360 Accessing Our World From Every Angle CJASN Clinical Journal of the American Society of Nephrology

ASN Journal Disclosure Form

As per ASN journal policy, I have disclosed any financial relationship or commitment held by myself and/or my spouse/partner in the past 36 months as included below. I have listed my Current Employer below to indicate there is a relationship requiring disclosure. If no relationship exists, my Current Employer is not listed.

J. Chan reports the following:
Employer: The Chinese University of Hong Kong; Consultancy: JCNC reported receiving grants (through institutions) and/or honoraria for consultancy or giving lectures from Applied Therapeutics, AstraZeneca, Bayer, Boehringer Ingelheim, Celltrion, Eli Lilly, Hua Medicine, Powder Pharmaceuticals, Roche, Merck, MSD, Pfizer, Sanofi, Servier, Viatris and Zuelig Pharma.; Ownership Interest: Cofounder of GemVCare, a biotech company for precision medicine with partial support from Hong Kong Government; Research Funding: JCNC reported receiving grants (through institutions) and/or honoraria for consultancy or giving lectures from Applied Therapeutics, AstraZeneca, Bayer, Boehringer Ingelheim, Celltrion, Eli Lilly, Hua Medicine, Powder Pharmaceuticals, Roche, Merck, MSD, Pfizer, Sanofi, Servier, Viatris and Zuelig Pharma.; Honoraria: JCNC reported receiving grants (through institutions) and/or honoraria for consultancy or giving lectures from Applied Therapeutics, AstraZeneca, Bayer, Boehringer Ingelheim, Celltrion, Eli Lilly, Hua Medicine, Powder Pharmaceuticals, Roche, Merck, MSD, Pfizer, Sanofi, Servier, Viatris and Zuelig Pharma.; Patents or Royalties: Co-inventor of patents for using biogenetic markers to predict, prevent and personalize care for diabetes and its complications through application by the Chinese University of Hong Kong; Advisory or Leadership Role: CEO (probono) and board member of Asia Diabetes Foundation governed by the Chinese University of Hong Kong Foundation; and Speakers Bureau: JCNC reported receiving grants (through institutions) and/or honoraria for consultancy or giving lectures from Applied Therapeutics, AstraZeneca, Bayer, Boehringer Ingelheim, Celltrion, Eli Lilly, Hua Medicine, Powder Pharmaceuticals, Roche, Merck, MSD, Pfizer, Sanofi, Servier, Viatris and Zuelig Pharma.

I understand that the information above will be published within the journal article, if accepted, and that failure to comply and/or to accurately and completely report the potential financial conflicts of interest could lead to the following: 1) Prior to publication, article rejection, or 2) Post-publication, sanctions ranging from, but not limited to, issuing a correction, reporting the inaccurate information to the authors' institution, banning authors from submitting work to ASN journals for varying lengths of time, and/or retraction of the published work.

Name: Juliana CN Chan
Manuscript ID: JASN-2023-001601R1
Manuscript Title: Set7 Methyltransferase Regulates the Phenotypic Switch in Diabetic Glomerular Endothelial Cells,
Date of Completion: March 8, 2024
Disclosure Updated Date: March 8, 2024

ASN American Society of Nephrology
JASN Journal of the American Society of Nephrology
KIDNEY360 Accessing Our World From Every Angle
CJASN Clinical Journal of the American Society of Nephrology

ASN Journal Disclosure Form

As per ASN journal policy, I have disclosed any financial relationship or commitment held by myself and/or my spouse/partner in the past 36 months as included below. I have listed my Current Employer below to indicate there is a relationship requiring disclosure. If no relationship exists, my Current Employer is not listed.

B. Chow has nothing to disclose.

I understand that the information above will be published within the journal article, if accepted, and that failure to comply and/or to accurately and completely report the potential financial conflicts of interest could lead to the following: 1) Prior to publication, article rejection, or 2) Post-publication, sanctions ranging from, but not limited to, issuing a correction, reporting the inaccurate information to the authors' institution, banning authors from submitting work to ASN journals for varying lengths of time, and/or retraction of the published work.

Name: Bryna Chow
Manuscript ID: JASN-2023-001601R1
Manuscript Title: Set7 methyltransferase regulates the phenotypic switch in diabetic glomerular endothelial cells
Date of Completion: February 22, 2024
Disclosure Updated Date: February 22, 2024

ASN Journal Disclosure Form

As per ASN journal policy, I have disclosed any financial relationship or commitment held by myself and/or my spouse/partner in the past 36 months as included below. I have listed my Current Employer below to indicate there is a relationship requiring disclosure. If no relationship exists, my Current Employer is not listed.

M. Cooper reports the following:
Employer: Monash University; Consultancy: AstraZeneca; Boehringer Ingelheim;; Eli Lilly; NovoNordisk;; Honoraria: Boehringer Ingelheim; Astra Zeneca; and Speakers Bureau: Boehringer Ingelheim.

I understand that the information above will be published within the journal article, if accepted, and that failure to comply and/or to accurately and completely report the potential financial conflicts of interest could lead to the following: 1) Prior to publication, article rejection, or 2) Post-publication, sanctions ranging from, but not limited to, issuing a correction, reporting the inaccurate information to the authors' institution, banning authors from submitting work to ASN journals for varying lengths of time, and/or retraction of the published work.

Name: Mark E. Cooper
Manuscript ID: JASN-2023-001601R1
Manuscript Title: Set7
Date of Completion: March 14, 2024
Disclosure Updated Date: March 5, 2024

ASN American Society of Nephrology · JASN Journal of the American Society of Nephrology · KIDNEY360 Accessing Our World From Every Angle · CJASN Clinical Journal of the American Society of Nephrology

ASN Journal Disclosure Form

As per ASN journal policy, I have disclosed any financial relationship or commitment held by myself and/or my spouse/partner in the past 36 months as included below. I have listed my Current Employer below to indicate there is a relationship requiring disclosure. If no relationship exists, my Current Employer is not listed.

S. El-Osta reports the following:
Patents or Royalties: Central Clinical School, Monash University

I understand that the information above will be published within the journal article, if accepted, and that failure to comply and/or to accurately and completely report the potential financial conflicts of interest could lead to the following: 1) Prior to publication, article rejection, or 2) Post-publication, sanctions ranging from, but not limited to, issuing a correction, reporting the inaccurate information to the authors' institution, banning authors from submitting work to ASN journals for varying lengths of time, and/or retraction of the published work.

Name: Sam El-Osta
Manuscript ID: JASN-2023-001601R1
Manuscript Title: Set7 methyltransferase regulates the phenotypic switch in diabetic glomerular endothelial cells
Date of Completion: February 22, 2024
Disclosure Updated Date: February 22, 2024

ASN American Society of Nephrology | JASN Journal of the American Society of Nephrology | KIDNEY360 Accessing Our World From Every Angle | CJASN Clinical Journal of the American Society of Nephrology

ASN Journal Disclosure Form

As per ASN journal policy, I have disclosed any financial relationship or commitment held by myself and/or my spouse/partner in the past 36 months as included below. I have listed my Current Employer below to indicate there is a relationship requiring disclosure. If no relationship exists, my Current Employer is not listed.

Y. Huang has nothing to disclose.

I understand that the information above will be published within the journal article, if accepted, and that failure to comply and/or to accurately and completely report the potential financial conflicts of interest could lead to the following: 1) Prior to publication, article rejection, or 2) Post-publication, sanctions ranging from, but not limited to, issuing a correction, reporting the inaccurate information to the authors' institution, banning authors from submitting work to ASN journals for varying lengths of time, and/or retraction of the published work.

Name: Yu Huang
Manuscript ID: JASN-2023-001601R1
Manuscript Title: Set7 Methyltransferase Regulates the Phenotypic Switch in Diabetic Glomerular Endothelial Cells
Date of Completion: March 4, 2024
Disclosure Updated Date: March 4, 2024

ASN Journal Disclosure Form

As per ASN journal policy, I have disclosed any financial relationship or commitment held by myself and/or my spouse/partner in the past 36 months as included below. I have listed my Current Employer below to indicate there is a relationship requiring disclosure. If no relationship exists, my Current Employer is not listed.

K. Jandeleit-Dahm reports the following:
Employer: Monash University; Research Funding: Boehringer Ingelheim; EFSD/Eli Lilly, Astra Zeneca; Honoraria: Boehringer Ingelheim; and Advisory or Leadership Role: Diabetes, Clinical Science.

I understand that the information above will be published within the journal article, if accepted, and that failure to comply and/or to accurately and completely report the potential financial conflicts of interest could lead to the following: 1) Prior to publication, article rejection, or 2) Post-publication, sanctions ranging from, but not limited to, issuing a correction, reporting the inaccurate information to the authors' institution, banning authors from submitting work to ASN journals for varying lengths of time, and/or retraction of the published work.

Name: Karin Jandeleit-Dahm
Manuscript ID: JASN-2023-001601R1
Manuscript Title: Set7 Methyltransferase Regulates the Phenotypic Switch in Diabetic Glomerular Endothelial Cells
Date of Completion: March 14, 2024
Disclosure Updated Date: March 14, 2024

ASN American Society of Nephrology | JASN Journal of the American Society of Nephrology | KIDNEY360 Accessing Our World From Every Angle | CJASN Clinical Journal of the American Society of Nephrology

ASN Journal Disclosure Form

As per ASN journal policy, I have disclosed any financial relationship or commitment held by myself and/or my spouse/partner in the past 36 months as included below. I have listed my Current Employer below to indicate there is a relationship requiring disclosure. If no relationship exists, my Current Employer is not listed.

H. Kaipananickal reports the following:
Employer: Baker Heart and Diabetes Institute

I understand that the information above will be published within the journal article, if accepted, and that failure to comply and/or to accurately and completely report the potential financial conflicts of interest could lead to the following: 1) Prior to publication, article rejection, or 2) Post-publication, sanctions ranging from, but not limited to, issuing a correction, reporting the inaccurate information to the authors' institution, banning authors from submitting work to ASN journals for varying lengths of time, and/or retraction of the published work.

Name: Harikrishnan Kaipananickal
Manuscript ID: JASN-2023-001601R1
Manuscript Title: Set7 methyltransferase regulates the phenotypic switch in diabetic glomerular endothelial cells
Date of Completion: February 23, 2024
Disclosure Updated Date: February 23, 2024

ASN American Society of Nephrology | JASN Journal of the American Society of Nephrology | KIDNEY360 Accessing Our World From Every Angle | CJASN Clinical Journal of the American Society of Nephrology

ASN Journal Disclosure Form

As per ASN journal policy, I have disclosed any financial relationship or commitment held by myself and/or my spouse/partner in the past 36 months as included below. I have listed my Current Employer below to indicate there is a relationship requiring disclosure. If no relationship exists, my Current Employer is not listed.

T. Karagiannis reports the following:
Employer: Baker Heart and Diabetes Institute

I understand that the information above will be published within the journal article, if accepted, and that failure to comply and/or to accurately and completely report the potential financial conflicts of interest could lead to the following: 1) Prior to publication, article rejection, or 2) Post-publication, sanctions ranging from, but not limited to, issuing a correction, reporting the inaccurate information to the authors' institution, banning authors from submitting work to ASN journals for varying lengths of time, and/or retraction of the published work.

Name: Tom C Karagiannis
Manuscript ID: JASN-2023-001601R1
Manuscript Title: Set7 methyltransferase regulates the phenotypic switch in diabetic glomerular endothelial cells
Date of Completion: February 22, 2024
Disclosure Updated Date: February 22, 2024

ASN American Society of Nephrology | JASN Journal of the American Society of Nephrology | KIDNEY360 Accessing Our World From Every Angle | CJASN Clinical Journal of the American Society of Nephrology

ASN Journal Disclosure Form

As per ASN journal policy, I have disclosed any financial relationship or commitment held by myself and/or my spouse/partner in the past 36 months as included below. I have listed my Current Employer below to indicate there is a relationship requiring disclosure. If no relationship exists, my Current Employer is not listed.

I. Khurana has nothing to disclose.

I understand that the information above will be published within the journal article, if accepted, and that failure to comply and/or to accurately and completely report the potential financial conflicts of interest could lead to the following: 1) Prior to publication, article rejection, or 2) Post-publication, sanctions ranging from, but not limited to, issuing a correction, reporting the inaccurate information to the authors' institution, banning authors from submitting work to ASN journals for varying lengths of time, and/or retraction of the published work.

Name: Ishant Khurana
Manuscript ID: JASN-2023-001601R1
Manuscript Title: Set7 Methyltransferase Regulates the Phenotypic Switch in Diabetic Glomerular Endothelial Cells
Date of Completion: March 6, 2024
Disclosure Updated Date: March 6, 2024

ASN American Society of Nephrology | JASN Journal of the American Society of Nephrology | KIDNEY360 Accessing Our World From Every Angle | CJASN Clinical Journal of the American Society of Nephrology

ASN Journal Disclosure Form

As per ASN journal policy, I have disclosed any financial relationship or commitment held by myself and/or my spouse/partner in the past 36 months as included below. I have listed my Current Employer below to indicate there is a relationship requiring disclosure. If no relationship exists, my Current Employer is not listed.

R. Ma reports the following:
Consultancy: AstraZeneca, Boehringer Ingelheim, Daiichi Sankyo, Eli Lilly, Kyowa Kirin, Merck, MSD.; Research Funding: Novo Nordisk, Roche Diagnostics (Hong Kong) Limited.; and Patents or Royalties: I hold patents, outside of the submitted work, filed through my academic institution, relating to genetic and other biomarkers for diabetes and related complications. Some of these have been licensed by a technology start-up, GemVCare.

I understand that the information above will be published within the journal article, if accepted, and that failure to comply and/or to accurately and completely report the potential financial conflicts of interest could lead to the following: 1) Prior to publication, article rejection, or 2) Post-publication, sanctions ranging from, but not limited to, issuing a correction, reporting the inaccurate information to the authors' institution, banning authors from submitting work to ASN journals for varying lengths of time, and/or retraction of the published work.

Name: Ronald C. Ma
Manuscript ID: 2023-001601R1
Manuscript Title: Set7 Methyltransferase Regulates the Phenotypic Switch in Diabetic Glomerular Endothelial Cells
Date of Completion: March 5, 2024
Disclosure Updated Date: March 5, 2024

ASN Journal Disclosure Form

As per ASN journal policy, I have disclosed any financial relationship or commitment held by myself and/or my spouse/partner in the past 36 months as included below. I have listed my Current Employer below to indicate there is a relationship requiring disclosure. If no relationship exists, my Current Employer is not listed.

S. Maxwell has nothing to disclose.

I understand that the information above will be published within the journal article, if accepted, and that failure to comply and/or to accurately and completely report the potential financial conflicts of interest could lead to the following: 1) Prior to publication, article rejection, or 2) Post-publication, sanctions ranging from, but not limited to, issuing a correction, reporting the inaccurate information to the authors' institution, banning authors from submitting work to ASN journals for varying lengths of time, and/or retraction of the published work.

Name: Scott S Maxwell
Manuscript ID: JASN-2023-001601R1
Manuscript Title: Set7 methyltransferase regulates the phenotypic switch in diabetic glomerular endothelial cells
Date of Completion: February 22, 2024
Disclosure Updated Date: February 22, 2024

ASN American Society of Nephrology | JASN Journal of the American Society of Nephrology | KIDNEY360 Accessing Our World From Every Angle | CJASN Clinical Journal of the American Society of Nephrology

ASN Journal Disclosure Form

As per ASN journal policy, I have disclosed any financial relationship or commitment held by myself and/or my spouse/partner in the past 36 months as included below. I have listed my Current Employer below to indicate there is a relationship requiring disclosure. If no relationship exists, my Current Employer is not listed.

J. Okabe reports the following:
Employer: Baker Heart and Diabetes Institute; Monash University

I understand that the information above will be published within the journal article, if accepted, and that failure to comply and/or to accurately and completely report the potential financial conflicts of interest could lead to the following: 1) Prior to publication, article rejection, or 2) Post-publication, sanctions ranging from, but not limited to, issuing a correction, reporting the inaccurate information to the authors' institution, banning authors from submitting work to ASN journals for varying lengths of time, and/or retraction of the published work.

Name: Jun Okabe
Manuscript ID: JASN-2023-001601R1
Manuscript Title: Set7 methyltransferase regulates the phenotypic switch in diabetic glomerular endothelial cells
Date of Completion: February 22, 2024
Disclosure Updated Date: February 22, 2024

ASN American Society of Nephrology
JASN Journal of the American Society of Nephrology
KIDNEY360 Accessing Our World From Every Angle
CJASN Clinical Journal of the American Society of Nephrology

ASN Journal Disclosure Form

As per ASN journal policy, I have disclosed any financial relationship or commitment held by myself and/or my spouse/partner in the past 36 months as included below. I have listed my Current Employer below to indicate there is a relationship requiring disclosure. If no relationship exists, my Current Employer is not listed.

E. Pitsillou has nothing to disclose.

I understand that the information above will be published within the journal article, if accepted, and that failure to comply and/or to accurately and completely report the potential financial conflicts of interest could lead to the following: 1) Prior to publication, article rejection, or 2) Post-publication, sanctions ranging from, but not limited to, issuing a correction, reporting the inaccurate information to the authors' institution, banning authors from submitting work to ASN journals for varying lengths of time, and/or retraction of the published work.

Name: Eleni Pitsillou
Manuscript ID: JASN-2023-001601R1
Manuscript Title: Set7 methyltransferase regulates the phenotypic switch in diabetic glomerular endothelial cells
Date of Completion: February 22, 2024
Disclosure Updated Date: February 22, 2024

ASN American Society of Nephrology
JASN Journal of the American Society of Nephrology
KIDNEY360 Accessing Our World From Every Angle
CJASN Clinical Journal of the American Society of Nephrology

ASN Journal Disclosure Form

As per ASN journal policy, I have disclosed any financial relationship or commitment held by myself and/or my spouse/partner in the past 36 months as included below. I have listed my Current Employer below to indicate there is a relationship requiring disclosure. If no relationship exists, my Current Employer is not listed.

H. Rodriguez has nothing to disclose.

I understand that the information above will be published within the journal article, if accepted, and that failure to comply and/or to accurately and completely report the potential financial conflicts of interest could lead to the following: 1) Prior to publication, article rejection, or 2) Post-publication, sanctions ranging from, but not limited to, issuing a correction, reporting the inaccurate information to the authors' institution, banning authors from submitting work to ASN journals for varying lengths of time, and/or retraction of the published work.

Name: Hanah Rodriguez
Manuscript ID: JASN-2023-001601R1
Manuscript Title: Set7 methyltransferase regulates the phenotypic switch in diabetic glomerular endothelial cells
Date of Completion: March 1, 2024
Disclosure Updated Date: March 1, 2024
