# Supplementary material for: Set7 Methyltransferase and Phenotypic Switch in Diabetic Glomerular Endothelial Cells
Source: J Am Soc Nephrol. 2024 Apr 17;35(6):733–48. doi: 10.1681/ASN.0000000000000345 (PMC11164123; doi:10.1681/ASN.0000000000000345)
Supplement: Supplementary file 2 [file jasn-35-733-s002.pdf]

Supplemental File

Set7 methyltransferase and phenotypic switch in diabetic glomerular endothelial cells

Scott Maxwell[1,2*], Jun Okabe[1,2*], Harikrishnan Kaipananickal.[1,2,3], Hanah Rodriguez[1,2], Ishant Khurana[1,2], Keith Al-Hasani[1,2], Bryna S.M. Chow[2], Eleni Pitsillou[4], Tom C. Karagiannis[1,2,4], Karin Jandeleit-Dahm[2,5], Ronald CW Ma[6,7,8], Yu Huang[8,9,10], Juliana CN Chan[6,7,8], Mark E Cooper[2] and Assam El-Osta[1,2,3,6,7,9,10,11]

[1]Epigenetics in Human Health and Disease Laboratory, [2]Department of Diabetes, Central Clinical School, Monash University, Melbourne, Victoria, Australia. [3]Department of Clinical Pathology, The University of Melbourne, Parkville, Victoria, Australia. [4]School of Science, STEM College, RMIT University, Melbourne, Victoria, Australia. [5]Institute for Clinical Diabetology, Research Group Diabetic Nephropathy, German Diabetes Centre, Heinrich Heine University, Duesseldorf, Germany, [6]Department of Medicine and Therapeutics, The Chinese University of Hong Kong (CUHK), Hong Kong SAR, China. [7]Hong Kong Institute of Diabetes and Obesity, CUHK, Hong Kong SAR. [8]Li Ka Shing Institute of Health Sciences, CUHK, Hong Kong SAR, China. [9]School of Biomedical Sciences, CUHK, Hong Kong SAR, China. [10]Department of Biomedical Sciences, City University of Hong Kong, Hong Kong SAR, China. [11]University College Copenhagen, Faculty of Health, Department of Technology, Biomedical Laboratory Science, Copenhagen, Denmark.

*authors contributed equally

Corresponding Author Mailing Address, Baker Heart and Diabetes Institute, 75 Commercial Road, Melbourne 3004, Vic, Australia. Professor Assam El-Osta sam.el-osta@baker.edu.au

Table of Contents

Supplemental Materials and Methods	Page 3-9
Supplemental Table 1	Page 10
Supplemental Table 2	Page 11
Supplemental Figure 1	Page 12
Supplemental Figure 2	Page 13
Supplemental Figure 3	Page 14
Supplemental Figure 4	Page 15
Supplemental Figure 5	Page 16
Supplemental Figure 6	Page 17-18
Supplemental References	Page 19-20

SUPPLEMENTAL MATERIALS AND METHODS

Diabetic Mouse Model

The Set7 knockout (Set7$^{-/-}$) mice were generated by genOway (Lyon, France). The *Setd7* targeting vector was designed within the first MORN domain of exon 2 containing two loxP sites. Insertion of loxP sites was introduced 1.6kb upstream of exon 2 by the integration of a loxP-flanked neomycin cassette. A targeting vector containing loxP sites flanking exon 2 of the *Setd7* gene was integrated by homologous recombination in mouse embryonic stem cells. Recombinant clones were injected into mouse C57BL/6J strain blastocysts and implanted into pseudopregnant females. Once the construct was integrated into C57BL/6J background mice, it was crossed with a Cre/loxP expression recombinase mouse to create a constitutive Set7$^{-/-}$ mouse. Set7$^{-/-}$ mice were back-crossed with the ApoE$^{-/-}$ mouse strain [1] for 10 generations to generate double knout-out animals (Set7$^{-/-}$ApoE$^{-/-}$). Six-week-old male Set7$^{+/+}$ApoE$^{-/-}$ and Set7$^{-/-}$ApoE$^{-/-}$ mice were rendered diabetic by five daily IP injections of streptozotocin (Sigma-Aldrich, St. Louis, MO) at a dose of 55mg/kg. At 10 weeks after the induction of diabetes, animals were measured the levels of blood glucose and glycated haemoglobin (HbA1c), and metabolic parameters to confirm the progression of diabetes. HbA1c was measured using the Cobas b101 POC system (Roche Diagnostics, Switzerland). Albuminuria was determined in the urine using Mouse Albumin ELISA Quantitation Set (Bethyl Laboratories, Montgomery, TX). Creatinine was measured determined by a commercially available creatinine assay kit using the Cobas Integra 400 Plus computerized analyser (Roche Diagnostics). The urinary albumin excretion (UAE) and albumin-to-creatinine ratio (ACR) were calculated as previously described [2]. After 10 weeks, animals were euthanized by CO_2 overload. The kidneys were rapidly dissected and processed for subsequent analyses. All animal experiments were approved by the Alfred Medical Research and Education Precinct (AMREP) Animal Ethics Committee under project number E/1504/2014/B.

Histological Assessment

Paraffin tissue sections of kidney were used for Periodic-acid Schiff staining and immunohistochemical staining with goat anti-type IV collagen (Southern Biotechnology, Birmingham, AL) and rabbit anti-type I collagen (Biodesign International, Saco, ME) as previously described [1,2]. All digital quantification using Image-Pro Plus 6.0 (Media Cybernetics, Bethesda, MD) and assessments were performed in a blinded manner as described previously [2].

RNA Analyses

Samples were homogenized or lysed in TRIzol® (Life Technologies, Waltham, MA) and RNA was isolated using the Direct-zol™ RNA Mini prep kit (Zymo Research, Irvine, CA) according to the manufacturer's instructions. cDNA synthesis, qRT-PCR analysis of gene expression and RNA-sequencing were performed as previously described [3,4]. Primer sequences are described in the Supplementary Table 1. Barcoded RNA-seq libraries were generated from ribosomal RNA depleted total RNA using the NEBNext® rRNA Depletion Kit (Human/Mouse/Rat) (New England Biolabs, Ipswich, MA) in conjunction with the NEBNext® Ultra™ Directional RNA Library Prep Kit for Illumina® (New England Biolabs) using 1µg of input total RNA and following the manufacturer's instructions for depletion and library construction. RNA-seq libraries were sequenced using Illumina's Nova-Seq platform by Novogene (Singapore). Sequence reads underwent quality and adapter trimming using skewer. Trimmed reads were mapped to human genome build GRCh38 (Ensembl) using STAR aligner. Tags aligning to genes were counted using FeatureCounts with Ensembl annotations. Genes with less than 10 reads average across all samples were excluded from the analysis, and changes in gene expression determined using edgeR [5].

Single-Cell Sequencing

Preparation of renal cells from kidney tissue was processed individually at 4°C. Mouse kidney was collected and chilled in ice cold phosphate buffered saline (PBS) before dissection of cortex tissue.

The isolated cortex material was initially minced using a scalpel, then pressed through a 100-µm cell sieve (Corning, NY) using the plunger of a 1 mL syringe while rinsing material through with PBS. The homogenate collected in a 50 mL centrifuge tube (Corning) was spun at 250x*g* for 2 minutes and supernatant discarded. The pellet was resuspended in 20 mL ice-cold PBS and poured through a 70-µm cell sieve to capture glomeruli while allowing smaller structures to pass. The "glomerular" fraction as caught by the sieve was transferred to a 15 mL tube using a Pasteur pipette and a volume of cold PBS up to 15 mL. The recovered fraction was then centrifuged at 200x*g* for 5 minutes, after which the supernatant was carefully removed to avoid disturbing pelleted material. The glomerular pellet was resuspended in 1 mL of digestion buffer (10 mg protease from *Bacillus licheniformis* (Sigma, P5380), 10uL DNase (Zymo Research, E1011), 0.5mM EDTA in 1mL PBS), and allowed to digest for 13 minutes at 4˚ C with slow rotation. To stop further digestion, 1 mL of PBS with 20 % Fetal Bovine Serum (FBS) was added. To aid dissociation, the mixture was additionally triturated using a 1000 µL pipette very gently. Triturated material was passed through a 40-µm cell sieve and collected in a 50 mL tube by rinsing through with 2% FBS in PBS. Material that did not pass through the 40-µm cell sieve was further triturated several times until no remaining clumps were left on the sieve. Immediately prior to starting single cell capture, the dissociated glomerular fraction was centrifuged at 200x*g* for 5 minutes with washed at least 5 times (200x*g*, 1 mL filtered PBS with 0.01% Bovine serum albumin) to reduce free RNA in solution. Single-cell capture and transcriptomic profiling were performed using droplet based (Drop-Seq) technology and single cell microfluidics platform from Dolomite Bio (Royston, UK), according to Dropseq-Protocol-v.-3.1-Dec-2015 [6] with minor modifications (Terra PCR Direct Polymerase Mix was used for cDNA amplification using 4 cycles in the first stage, and 11 cycles in stage two). Barcoded libraries were generated using the Nextera XT DNA Library Preparation Kit (Illumina) in house and sequenced using Illumina Hiseq paired-end 150-cycle runs by Novogene (Singapore).

Single-Cell Data Analysis

Raw single-cell data was demultiplexed and counted using Drop-Seq tools version 2.1 [6] before clustering and downstream analysis using the R package Seurat version 3 [7] . Quality filters included removal of cells with detection of less than 400 or more than 3000 genes, and cells with transcriptomes having more than 20% mitochondrial genes. Clustering was performed using Seurat functions RunTSNE, FindNeighbors, and FindClusters with PCA reduction – PCA dimensions (dims = 1:22). To identify specific cell types, unique gene signatures were generated for each cluster using FindAllMarkers and comparison with known endogenous marker genes. Differential gene expression was calculated using the Seurat function FindMarkers function to compare groups, specifying Wilcoxon rank sum analysis with min.pct set to 0.25. Single cell data for each experimental group was visualised by t-distributed stochastic neighbour embedding (tSNE) following analysis with the R package Seurat. Cluster identities were designated according to expression of specific marker genes. To determine changes in cell cluster proportion between groups, raw cell counts were transformed and subject to analysis of variance (ANOVA) using propeller functions included with the R package Speckle [8].

Gene Set Enrichment Analysis

For single cell data, cluster idents were designated according to expression of specific marker genes, while differential gene expression between experimental groups was calculated using the Seurat function FindMarkers (default settings with wilcoxon rank sum analysis and min.pct set to 0.25). For bulk RNA-seq, differential expression was calculated using the R package Edger. Gene Set Enrichment Analysis (GSEA) was conducted using the R package Mitch [9], with examination of Reactome pathways database v7 (https://reactome.org/). All detected genes were used in Mitch GSEA analysis in which genes are ranked according to the specific contrast p-value and fold change direction obtained from FindMarkers or Edger. Mitch typically uses the equation where gene rank is

determined by: -log10('p-value') * sign('log-fold-change') enabling identification of gene sets with predominantly increased or decreased expression.

Protein Analysis

Protein extraction, immunoblotting analysis were performed as previously described [3,4] . Protein signals were quantified by Odyssey CLx image system (LI-COR Biotechnology, Lincoln, NE). Anti-SET7 (#2813, Cell Signaling Technology (CST), Danvers, MA; 1:2,000 dilution), anti-Di-Methyl-RPL29 Lys5 (#19495, CST, 1:2,000 dilution) and anti-GAPDH (#AM4300, Invitrogen, Waltham, MA; 1:10,000 dilution) were used as primary antibodies. Set7 histone methyltransferase activity assay was performed as previously described [4,10]

Renal Cell Culture

Immortalized human podocytes and glomerular endothelial cells were cultured and differentiated as previously described [11,12]. HK2 derived from human proximal tubular cells (PTCs) were cultured as described previously [13]. The human mono macrophage (MM) THP1 cells were maintained in RPMI 1640 medium supplemented with 10% FBS, 2 mM L-glutamine and antibiotics. Cells were cultured with or without 15 μM (R)-PFI-2 (PFI-2; Cayman Chemicals, Ann Arbor, MI) dissolved in DMSO for 24 hours before exposing them to 5.5mM or 25 mM D-glucose in the presence or absence of 5 ng/ml TGF-β1 (R&D Systems, Minneapolis, MN) for 48 hours at 37°C. Knockdown of Set7 was performed in GEN cells by MISSION shRNA expressing lentivirus vector (Sigma) as described previously [3]. Cells transduced with MISSION Non-target shRNA control vector (Sigma) was used as a control. Cellular and mitochondrial ROS levels in the cells were determined by DCFDA and MitoSOX fluorogenic probes (Life Technology), respectively. The fluorescent signals (Ex/Em settings of 483/530 for DCFDA; 570/630 for MitoSOX) were measured on CLARIOstar Plus (BMG Labtech, Germany).

Chromatin Immunoprecipitation (ChIP) assay

ChIP assay was performed as described previously[3,4]. Cells were cross-linked with 1% formaldehyde for 10 min and then quenched with 0.125M glycine for 10 min. Cell pellets were resuspended in SDS Lysis buffer (1% SDS, 10mM EDTA, 50mM Tris-HCl, pH 8.0 and proteinase inhibitor cocktail) and sonicated for 30 min at 30 sec intervals of on or off using a Bioruptor (Diagenode, Denville, NJ) to shear chromatin to lengths ranging between 150 - 250bp. 5ug of pre-cleared chromatin with Dynabeads protein A (Invitrogen) was immunoprecipitated using anti-H3K4me1(#39297, Active Motif, Carlsbad, CA), anti-H3K4me2(#07-030, Millipore, Burlington, MA), or normal rabbit IgG(#sc-2027, Santa Cruz, Dallas, TX) antibodies. Quantification of the immunoprecipitated DNA was performed by qPCR and data is represented as enrichment of immunoprecipitated DNA samples corrected against input DNA. Primer sequences are listed in the Supplementary Table 2.

Molecular docking analyses

The RCSB Protein Data Bank was used to obtain crystal structures of Set7 in complex with PFI-2 (PDB ID: 4JLG) and the histone H3 peptide (PDB ID: 1O9S). The crystal structure of Set7 (PDB ID: 1N6A) in complex with S-adenosyl-L-methionine (SAM) (PDB ID: 1N6A) was utilised for comparison. The SAM and S-adenosyl-L-homocysteine (SAH) cofactors were retained in the crystal structures. The water molecules, co-crystallised inhibitor, and histone H3 peptide were removed prior to molecular docking. Missing residues were subsequently modelled using the Chimera interface to Modeller [14,15]. Polar hydrogens were added to the crystal structures using PyMOL [16]. AutoDockTools-1.5.7 was used to prepare the proteins as macromolecules and the co-crystallised Set7 inhibitor, PFI-2, as a ligand [17]. For the inhibitor-bound structure of Set7 (PDB ID: 4JLG), the receptor grid was generated around PFI-2 and was 20 x 20 x 20 Å in size. Molecular docking was performed using AutoDock Vina at an exhaustiveness of 2048 [18].

The results were analysed using Maestro 13.2 and Visual Molecular Dynamics 1.9.3 [19,20]. The root-mean-square deviation (RMSD) between the co-crystallised and docked structures of PFI-2 was

calculated [20]. To evaluate conformational changes in the crystal structures of Set7 (PDB ID: 1N6A, 1O9S, and 4JLG), the proteins were aligned in Maestro 13.2 and the RMSD of each residue was calculated [20].

The histone H3 peptide was extracted from the crystal structure of Set7 (PDB ID: 1O9S) and imported into Maestro 13.2. The peptide was 10 amino acids in length and contained a methylated lysine residue (MLZ). For protein-peptide docking, the methylated residue was mutated to a standard lysine at position 4 (K4) [20]. The structure was imported into PyRx and energy minimised using the universal force field through Open Babel (v.2.2.3) [21,22]. Protein-peptide docking was performed to examine the binding characteristics of the histone H3 peptide in the absence (PDB ID: 1O9S) and presence of the inhibitor PFI-2 (PDB ID: 4JLG) using the HPEPDOCK 2.0 web server [23]. The predicted binding mode and interactions of the top-ranking peptide conformation were evaluated in Maestro 13.2.

Statistical Analysis

Data are represented as mean ± standard error of the mean (SEM). Statistical significance was determined using Student's t-tests, and one- or two-way ANOVA with Tukey's posthoc test for multiple comparisons as necessary in Graphpad Prism 7 (San Diego, CA). A *P*-value less than 0.05 was considered statistically significant.

Data Availability Statement

All data are presented in the article are in the main text or the Supplemental File are available as raw sequencing data in NCBI's Gene Expression Omnibus (GEO) database GSE158626.

Supplemental Table 1

Primer sequences used for quantitative RT-PCR assays

**Gene Name**	**Forward (5'-3')**	**Reverse (5'-3')**
ACOX1	GGGATTCCGCCAGCTTCAA	ATGTTCTCGATCTCTCGGCG
ANGPTL4	TGCAAGATGACCTCAGATGGAGG	AGAACTCGCCGTGGGGATCCC
BMPER	ACATGTCCAGGCTGTGTGTT	CATTGTGTCCTGCCTCCAGT
CDH5	CACCCAGACCAAGTACACAT	ATGGTGAAAGCGTCCTGGTA
COL4A1	CAATATGAAAACCGTAAAGTGCC TTATA	CAGCAAGTAGAGGTCAATGAAGCA
COL4A3	TCGCCATAGCCGTTCACAGCC	GCCCGGTGCCCTCAGAACCT
EDN1	AAGCCCTCCAGAGAGCGTTAT	CCGAAGGTCTGTCACCAATGT
H3F3	GGTGTCTTCAAAAAGGCCAA	GCGAGAAATTGCTCAGGACT
IGFBP5	TTCCGGCCCAAACACACCCG	AGGGGCCCTGCTCAGACTCC
LTV1	AGCTCCAGTTTCAGGACCTCG	CTCTCCTGTTGCCTTATTGGC
MDH2	TCGGCCCAGAACAATGCTAAA	GCGGCTTTGGTCTCGATGT
NDUFA13	GACTGGAAGGTGGGGGAGTC	CGTGTACCACATGAAGCCGT
NDUFB2	AGAAGAGGTGCTGGGTCACT	GTGAGGCTGAGTCTACACCTT
NHP2	CCTCACGCGGAAGCTCTAC	ACCCAGGTCCCTTTTTCTCCT
PDGFA	ACTCCTGGAGATAGACTCCGT	GGACAGCTTCCTCGATGCTT
PECAM1	GGAAAGCAGATACTCTAGAACGG	CTCGGAACATGGATGTCCTTC
PLAT	CGCCCAGCCAGGAAATCCAT	GCGCAGCCATGACTGATGTT
SET7	TACCGCACGGGTTCTGCACAG	CCTCCAGGGTGCTGCCATCAA
TAGLN	CTCATGCCATAGGAAGGACC	GTCCGAACCCAGACACAAGT
THBS2	TTGGCAAACCAGGAGCTCAG	TGGTGACCAGCTTGCGTG
TIMP3	ACCGAGGCTTCACCAAGATG	CCATCATAGACGCGACCTGT
VIM	AATGGCTCGTCACCTTCGTG	GGCAGAGAAATCCTGCTCTC
mApoE	TTCGGAAGGAGCTGACTGGC	CCACTCGAGCTGATCTGTCA
mH3f3	GAGCTCCAGCCGAAGGAGAAG	CAGTACCAGGCCTGTAACGATGAG
mIgfbp5	GAATCCGAACAAGGCCCC	ATCCTTTGCGGTCACAGTTG
mKim1	TGTTGAGAGTGACAGTGGTCTG	GACGTGTGGGAATCTCTGGT
mSet7	CGCTCAGCCACCAGGAGCAC	GTCCAGGTGCCCTTCCACGG

Supplemental Table 2

Primer sequences used for ChIP assay

**Gene Region**	**Forward (5'-3')**	**Reverse (5'-3')**
IGFBP5-R1	TGGGATAGACTCGGCTAGACA	GCTTTGCGCAGCAAGTGG
IGFBP5-R2	AGAAACACGCCTCCTTTCAC	GGTGTCCGTACCTGTCCTTC
IGFBP5-R3	GCTGTAGTCCCATCTTCCCC	AGGGCTCATGTCCAAAAGGT

Supplemental Figure 1

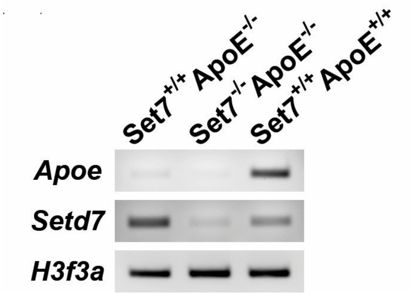

Supplemental Figure 1. mRNA expression in the kidney cortex derived from Set7$^{-/-}$ApoE$^{-/-}$ mice. mRNA expression of *Apoe* and *Setd7* in the mouse kidney cortex was determined by RT-PCR. *H3f3a* was used as loading control.

Supplemental Figure 2

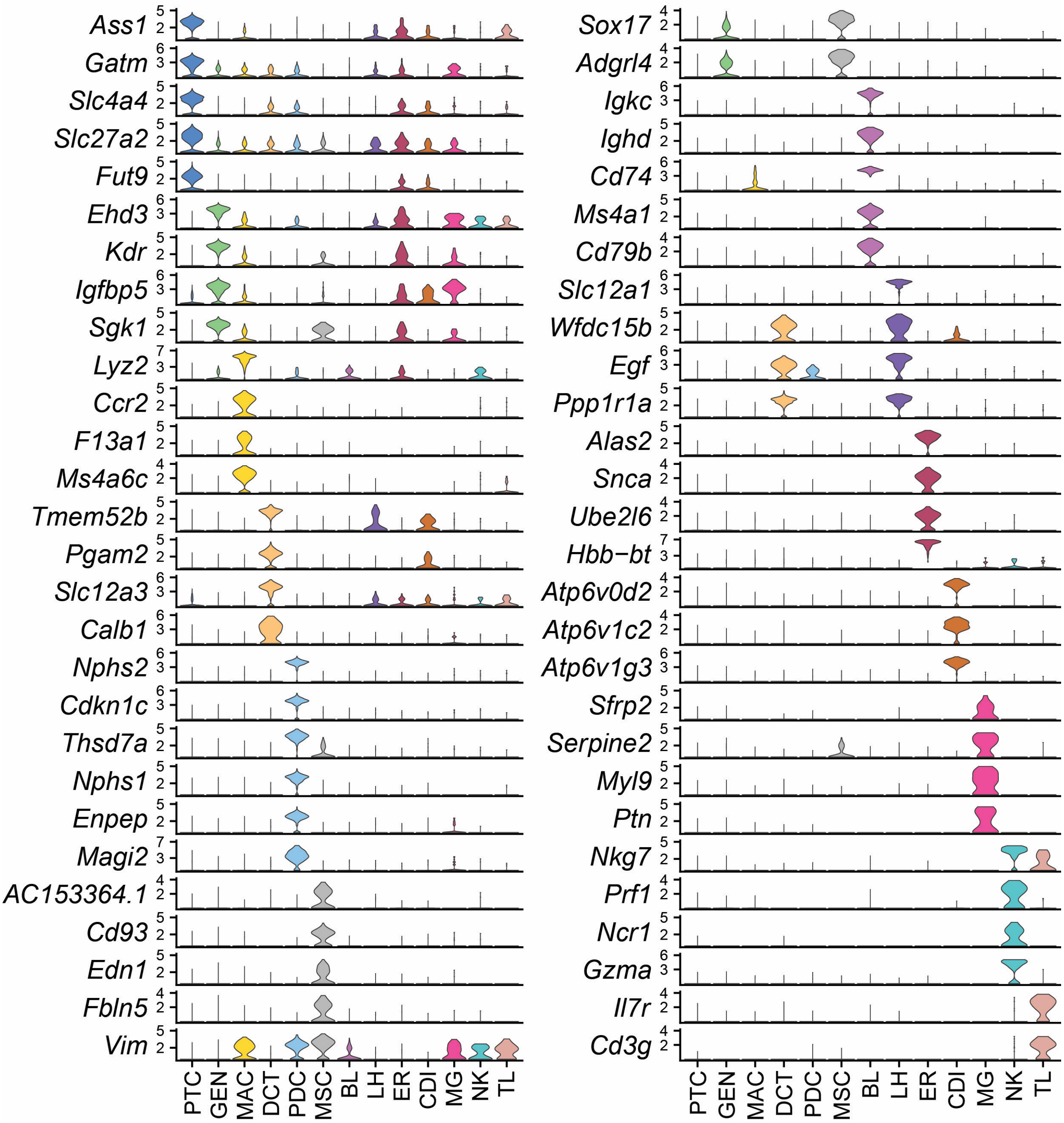

Supplemental Figure 2. Violin plots of cluster marker genes identified using the Seurat function 'FindAllMarkers' and represented as the following: PTC – proximal tubule cell, GEN – glomerular endothelial, MAC – macrophage, DCT – distal convoluted tubule, PDC – podocyte, MSC – mesenchymal, BL – B-lymphocyte, LH – Loop Henle, ER – erythroid, CDI – collecting duct intercalated, MG – mesangial, NK – natural killer and TL – T-lymphocyte.

Supplemental Figure 3

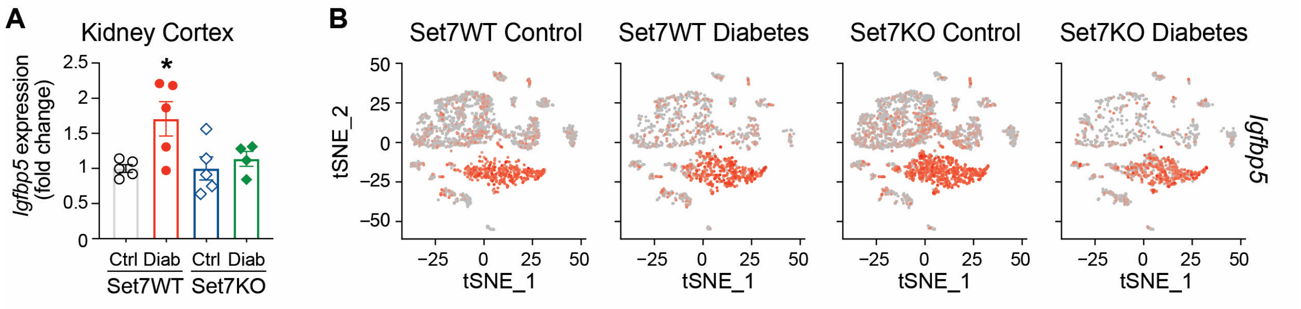

Supplemental Figure 3. *Igfbp5* mRNA expression patterns. (A) Increased *Igfbp5* expression in the diabetic kidney cortex was attenuated by Set7KO. The mRNA expression of *Igfbp5* in the kidney cortex was assessed by qRT-PCR. n=5 per group. Data are represented as mean ± SEM. * P<0.05 vs control Set7WT mice. (B) Distribution of *Igfbp5* expressions visualised by tSNE in each experimental group.

Supplemental Figure 4

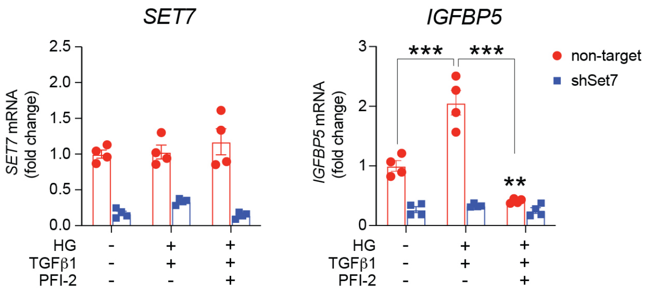

Supplemental Figure 4. Gene expression profiles in human shSet7 glomerular endothelial (GEN) cells treated with high glucose (HG), TGFβ1 and Set7 inhibitor (PFI-2). The expressions of SET7 and IGFBP5 in human non-target control or Set7 knock-down (shSet7) GEN cells were assessed by qRT-PCR. n=4 per group. Data are represented as mean ± SEM. ** P<0.01, *** P<0.001.

Supplemental Figure 5

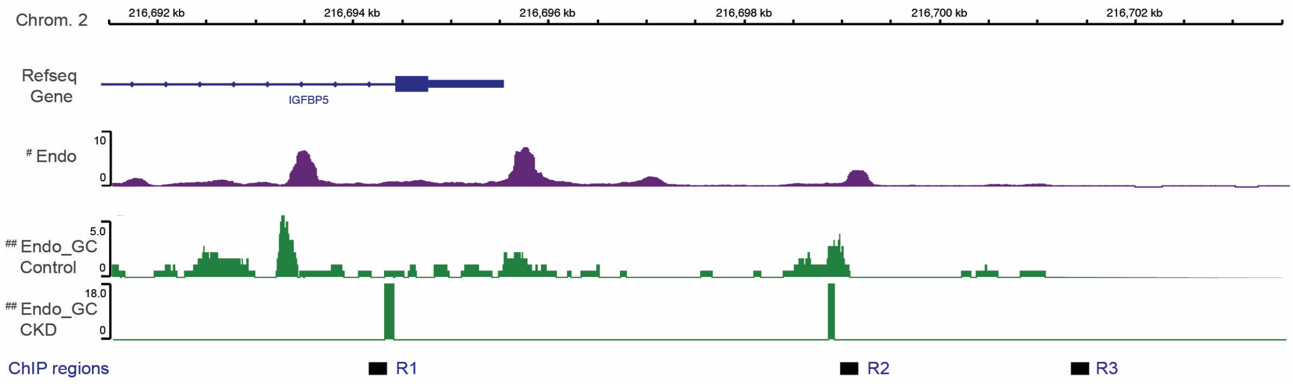

scATAC-seq data

Liu, H. et al., Nature Genetics, 2022, DOI: https://doi.org/10.1038/s41588-022-01097-w

Abedini, A . et al., BioRxiv, DOI: https://doi.org/10.1101/2022.10.24.513598

Supplemental Figure 5. Chromatin accessibility at the IGFBP5 promoter and enhancer region from publicly available scATAC-seq human kidney datasets. Glomerular endothelial scATAC-seq data also illustrated. Enrichment of H3K4 methylation determined at Regions R1, R2 and R3 were assessed by chromatin immunoprecipitation assay and described in Figure 6F and Figure 6G of the main article.

Supplemental Figure 6

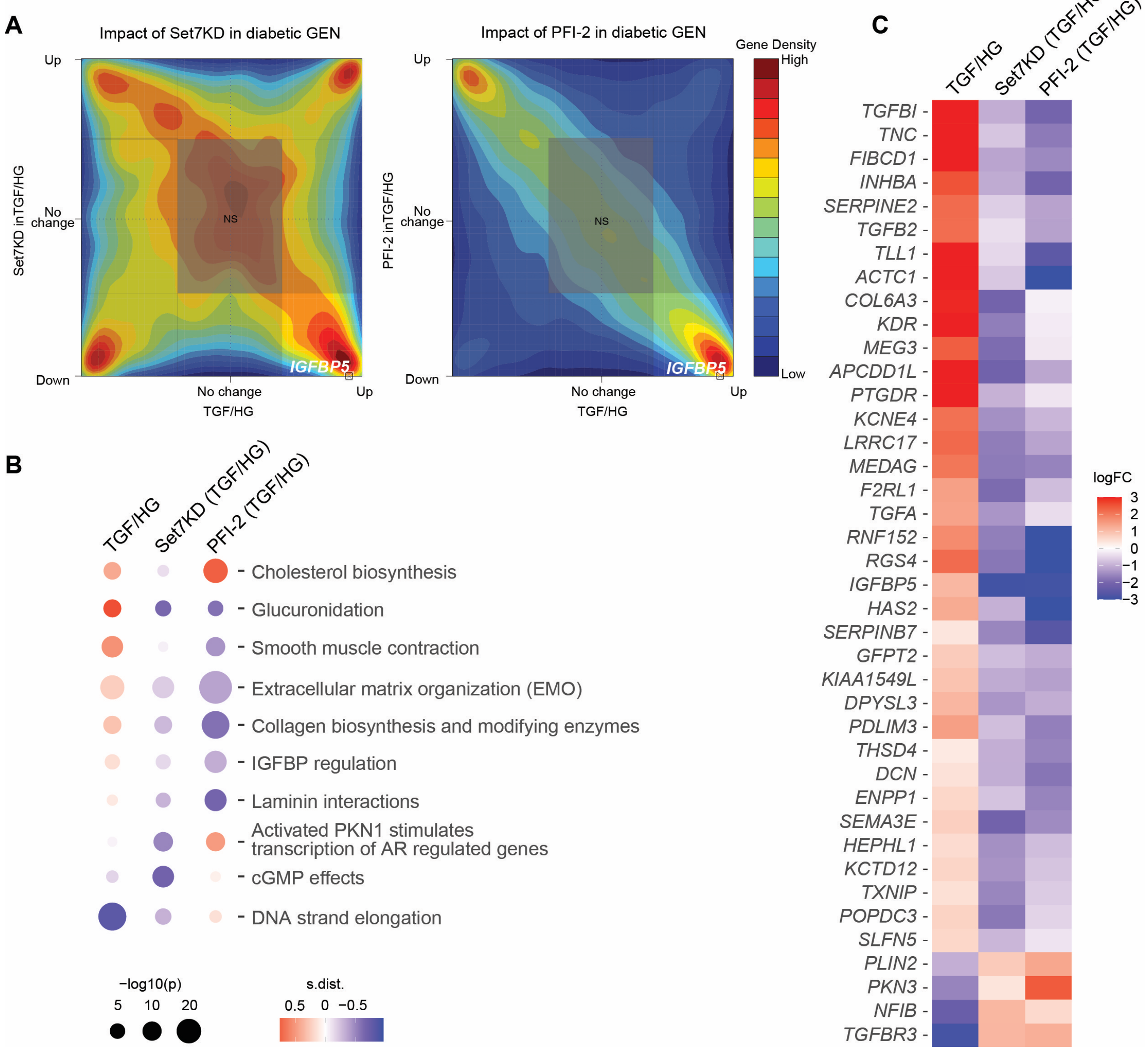

Supplemental Figure 6. RNA-seq analysis of human glomerular endothelial (GEN) cells stimulated with high glucose, TGFβ1 (TGF/HG) and Set7 inhibition (Set7KD or PFI-2). (A) The effect of Set7 inhibition in the diabetic glomerular endothelial (GEN) cells by two-dimensional contour plot. The abundance of gene transcripts that are differentially expressed between non-diabetic and diabetic GEN cells (TGF/HG, X-axis) was compared to the abundance of transcripts that are differentially expressed between diabetic GEN cells with Set7KD (left) or PFI-2 (right) (Y-axis). Red areas represent high gene density whereas blue areas represent a low number of genes. The location of a transcript along the axes represents whether its expression was up-regulated (Up), down-regulated (Down) or unchanged (UC) in that experimental condition. “Not significant” is shown as NS. (B) The Reactome pathways which are significantly changed by both TGF/HG and Set7 inhibitions in

TGF/HG. GSEA was used to determine enrichment of genes with transcriptional changes in response to TGF/HG and Set7KD or PFI-2 with the gene sets of reactome pathways. (C) Heatmap of top 40 genes which are significantly changed by TGF/HG and Set7 inhibitions (Set7KD or PFI-2) in TGF/HG. The genes which have p-value < 0.05 by both TGF/HG and Set7 inhibitions were clustered using hierarchical clustering.

Supplemental References

1. Lassila M, Seah KK, Allen TJ, et al. Accelerated nephropathy in diabetic apolipoprotein e-knockout mouse: role of advanced glycation end products. *J Am Soc Nephrol*. Aug 2004;15(8):2125-2138. doi:10.1097/01.ASN.0000133025.23732.46
2. Jha JC, Gray SP, Barit D, et al. Genetic targeting or pharmacologic inhibition of NADPH oxidase nox4 provides renoprotection in long-term diabetic nephropathy. *J Am Soc Nephrol*. Jun 2014;25(6):1237-1254. doi:10.1681/ASN.2013070810
3. Okabe J, Orlowski C, Balcerczyk A, et al. Distinguishing hyperglycemic changes by Set7 in vascular endothelial cells. *Circ Res*. Apr 13 2012;110(8):1067-1076. doi:10.1161/CIRCRESAHA.112.266171
4. Tuano NK, Okabe J, Ziemann M, Cooper ME, El-Osta A. Set7 mediated interactions regulate transcriptional networks in embryonic stem cells. *Nucleic Acids Res*. Nov 2 2016;44(19):9206-9217. doi:10.1093/nar/gkw621
5. Robinson MD, McCarthy DJ, Smyth GK. edgeR: a Bioconductor package for differential expression analysis of digital gene expression data. *Bioinformatics*. Jan 1 2010;26(1):139-140. doi:10.1093/bioinformatics/btp616
6. Macosko EZ, Basu A, Satija R, et al. Highly Parallel Genome-wide Expression Profiling of Individual Cells Using Nanoliter Droplets. *Cell*. May 21 2015;161(5):1202-1214. doi:10.1016/j.cell.2015.05.002
7. Stuart T, Butler A, Hoffman P, et al. Comprehensive Integration of Single-Cell Data. *Cell*. Jun 13 2019;177(7):1888-1902 e1821. doi:10.1016/j.cell.2019.05.031
8. Phipson B, Sim CB, Porrello ER, Hewitt AW, Powell J, Oshlack A. propeller: testing for differences in cell type proportions in single cell data. *Bioinformatics*. Oct 14 2022;38(20):4720-4726. doi:10.1093/bioinformatics/btac582
9. Kaspi A, Ziemann M. mitch: multi-contrast pathway enrichment for multi-omics and single-cell profiling data. *BMC Genomics*. Jun 29 2020;21(1):447. doi:10.1186/s12864-020-06856-9
10. Okabe J, Fernandez AZ, Ziemann M, Keating ST, Balcerczyk A, El-Osta A. Endothelial transcriptome in response to pharmacological methyltransferase inhibition. *ChemMedChem*. Aug 2014;9(8):1755-1762. doi:10.1002/cmdc.201402091
11. Saleem MA, O'Hare MJ, Reiser J, et al. A conditionally immortalized human podocyte cell line demonstrating nephrin and podocin expression. *J Am Soc Nephrol*. Mar 2002;13(3):630-638.
12. Satchell SC, Tasman CH, Singh A, et al. Conditionally immortalized human glomerular endothelial cells expressing fenestrations in response to VEGF. *Kidney Int*. May 2006;69(9):1633-1640. doi:10.1038/sj.ki.5000277
13. Ryan MJ, Johnson G, Kirk J, Fuerstenberg SM, Zager RA, Torok-Storb B. HK-2: an immortalized proximal tubule epithelial cell line from normal adult human kidney. *Kidney Int*. Jan 1994;45(1):48-57. doi:10.1038/ki.1994.6
14. Pettersen EF, Goddard TD, Huang CC, et al. UCSF Chimera--a visualization system for exploratory research and analysis. *J Comput Chem*. Oct 2004;25(13):1605-1612. doi:10.1002/jcc.20084
15. Sali A, Blundell TL. Comparative protein modelling by satisfaction of spatial restraints. *J Mol Biol*. Dec 5 1993;234(3):779-815. doi:10.1006/jmbi.1993.1626
16. Schrodinger L. The PyMOL molecular graphics system. Version. 1, 8. 2015;
17. Morris GM, Huey R, Lindstrom W, et al. AutoDock4 and AutoDockTools4: Automated docking with selective receptor flexibility. *J Comput Chem*. Dec 2009;30(16):2785-2791. doi:10.1002/jcc.21256
18. Trott O, Olson AJ. AutoDock Vina: improving the speed and accuracy of docking with a new scoring function, efficient optimization, and multithreading. *J Comput Chem*. Jan 30 2010;31(2):455-461. doi:10.1002/jcc.21334
19. Humphrey W, Dalke A, Schulten K. VMD: visual molecular dynamics. *J Mol Graph*. Feb 1996;14(1):33-38, 27-38. doi:10.1016/0263-7855(96)00018-5

20. Schrödinger L. Schrödinger Release 2022-2, Maestro. 2022;
21. Dallakyan S, Olson AJ. Small-molecule library screening by docking with PyRx. *Methods Mol Biol*. 2015;1263:243-250. doi:10.1007/978-1-4939-2269-7_19
22. O'Boyle NM, Banck M, James CA, Morley C, Vandermeersch T, Hutchison GR. Open Babel: An open chemical toolbox. *J Cheminform*. Oct 7 2011;3:33. doi:10.1186/1758-2946-3-33
23. Zhou P, Jin B, Li H, Huang SY. HPEPDOCK: a web server for blind peptide-protein docking based on a hierarchical algorithm. *Nucleic Acids Res*. Jul 2 2018;46(W1):W443-W450. doi:10.1093/nar/gky357
